# Metabolic Engineering for Enhanced Medium Chain Omega Hydroxy Fatty Acid Production in *Escherichia coli*

**DOI:** 10.3389/fmicb.2018.00139

**Published:** 2018-02-07

**Authors:** Kang Xiao, Xiu-Hong Yue, Wen-Chao Chen, Xue-Rong Zhou, Lian Wang, Lin Xu, Feng-Hong Huang, Xia Wan

**Affiliations:** ^1^Oil Crops Research Institute of the Chinese Academy of Agricultural Sciences, Wuhan, China; ^2^Key Laboratory of Biology and Genetic Improvement of Oil Crops, Ministry of Agriculture, Wuhan, China; ^3^Oil Crops and Lipids Process Technology National and Local Joint Engineering Laboratory, Wuhan, China; ^4^Hubei Key Laboratory of Lipid Chemistry and Nutrition, Wuhan, China; ^5^Agriculture and Food, Commonwealth Scientific and Industrial Research Organisation (CSIRO), Canberra, ACT, Australia

**Keywords:** *Escherichia coli*, medium chain fatty acid, hydroxy fatty acid, acyl-ACP thioesterase, fatty acid metabolism regulator

## Abstract

Medium chain hydroxy fatty acids (HFAs) at ω-1, 2, or 3 positions (ω-1/2/3) are rare in nature but are attractive due to their potential applications in industry. They can be metabolically engineered in *Escherichia coli*, however, the current yield is low. In this study, metabolic engineering with P450_BM3_ monooxygenase was applied to regulate both the chain length and sub-terminal position of HFA products in *E. coli*, leading to increased yield. Five acyl-acyl carrier protein thioesterases from plants and bacteria were first evaluated for regulating the chain length of fatty acids. Co-expression of the selected thioesterase gene *CcFatB1* with a fatty acid metabolism regulator *fadR* and monooxygenase *P450*_*BM*3_ boosted the production of HFAs especially ω-3-OH-C14:1, in both the wild type and *fadD* deficient strain. Supplementing renewable glycerol to reduce the usage of glucose as a carbon source further increased the HFAs production to 144 mg/L, representing the highest titer of such HFAs obtained in *E. coli* under the comparable conditions. This study illustrated an improved metabolic strategy for medium chain ω-1/2/3 HFAs production in *E. coli*. In addition, the produced HFAs were mostly secreted into culture media, which eased its recovery.

## Introduction

Hydroxy fatty acids (HFAs) are widely found in plants, animals, and microorganisms, although rare in medium-chain length (C8-C14). Their physiological roles, including anti-biotic, anti-inflammatory, and anti-diabetic effects, have been uncovered recently. In particular, the position of hydroxyl groups in the fatty acyl chain shows a key effect on the activity against certain plant pathogenic fungi. Branched chain HFAs generated by gut microbes are demonstrated to affect human health (Hou, 2009; Kishino et al., 2013; Sakurama et al., 2014; Yore et al., 2014). Compared to non-hydroxylated fatty acids, HFAs have higher reactivity, solvent miscibility, stability, and viscosity. Therefore, HFAs have been widely used in food, chemical, pharmaceutical, cosmetic industries, and as synthetic precursors (Yang et al., 2014; Meesapyodsuk et al., 2015; Bowen et al., 2016). Omega HFAs (ω-HFAs) are fatty acids with a hydroxyl group near the methyl end, or omega end. The hydroxylation at different positions of the fatty acid chain is needed for different chemical industries. For example, ω-HFAs can be used as renewable starting materials for the synthesis of polymers, with high resistance to heat or chemicals, flexibility, biocompatibility, and non-toxicity (Seo et al., 2015; Bowen et al., 2016). Meanwhile, the geometry of sub-terminal ω-1/2/3 HFA combined with different chain length provides diversity of characteristics for polymer synthesis from HFA, generating potential for wide range of commercial use.

Despite the wide applications of HFAs, direct synthesis of desired specific hydroxylation is limited and requires harsh reaction conditions. Enzymatic hydroxylation is an alternative approach due to its specificity for the positions to be hydroxylated. In general, four types of enzymes are involved in the production of diverse HFAs in nature, including cytochrome P450 monooxygenase, hydratase, hydroxylase, and lipoxygenase. P450 monooxygenases are classified into α-hydroxylase (carboxyl-terminal hydroxylase) and ω-hydroxylase (terminal or sub-terminal hydroxylase). Several P450 monooxygenases have been recently characterized with distinct substrate specificities from plant, yeast, and bacterium. Hydratases catalyze the hydration of a specific double bond. They are only found in microbes including *Stenotrophnomonas, Lactobacillus, Pediococcus, Selenomonas*, and *Enterococcus* (Hirata et al., 2015; Takeuchi et al., 2015, 2016). Hydroxylases introduce a hydroxyl group at single bond of fatty acid. Δ12-hydroxylases, for example, have been identified from plants *Ricinus communis, Lesquerella fendleri, Lesquerella lindheimeri*, or *Hiptage benghalensis*, and fungus *Claviceps purpurea* (Mavraganis et al., 2010; Zhou et al., 2013; Kim and Chen, 2015; Lee et al., 2015), which introduce a hydroxyl group at Δ12 position of oleic acid. Lipoxygenases use polyunsaturated fatty acids as substrates to form corresponding HFAs (Kim and Oh, 2013). Among those P450 monooxygenases, P450_BM3_ from *Bacillus megaterium* is the first one that has been characterized as a self-sufficient enzyme. It transfers electrons from NADPH during its oxidation as it contains a flavin reductase domain as a redox-partner. Such self-sufficient P450 enzymes are suitable as efficient and yet simple catalytic system due to their independence of an additional reductase enzyme and high turnover numbers (Narhi and Fulco, 1986). P450_BM3_ has broad substrate specificity, in addition to fatty acids, hydroxylating alkanols and alkylamides at sub-terminal positions as well. Purified P450_BM3_ hydroxylates fatty acids with chain length ranging from C12 to C18, and exhibits the highest specificity for C14:0, followed by C15:0 and C12:0, whereas, C16:0, C17:0, and C18:0 are used with the least efficiency (Schneider et al., 1998; Hilker et al., 2008). Three amino acids in P450_BM3_, R47, Y51, and F87, are crucial for substrate binding (Li and Poulos, 1999). Unlike fungal fatty acid hydroxylases, only sub-terminal positions are hydroxylated by the action of P450_BM3_.

Although considerable effort has been made in engineering of oil crops or microbes for producing HFAs, there has been limited success in the production of medium chain fatty acid (MCFA) sub-terminal HFAs (Meesapyodsuk et al., 2015; Bowen et al., 2016). Different strategies have been applied to increase MCFA sub-terminal or terminal HFAs production. Examples include overexpression of acyl-acyl carrier protein (acyl-ACP) thioesterase (or TE) to provide more MCFAs as substrates in *E. coli*, reducing fatty acid turnover, overexpression of NAD^+^ dependent formate dehydrogenase to produce cofactor NADH as well as optimization of fermentation process (Wang et al., 2012; Sung et al., 2015; Cao et al., 2016).

Since *E. coli* uses a Type II fatty acid biosynthesis, targeted chemicals of various chain lengths including MCFAs can be synthesized. In general, chain length is determined by 3-ketoacyl-ACP synthases and a variety of acyltransferases in *E. coli*. However, several plant TEs are able to regulate the chain length of *E. coli* fatty acid biosynthesis end products by hydrolyzing the acyl-ACP intermediates and releasing FFA. Of them, a few plant TEs (designated as FatBs) show preference for medium chain acyl-ACPs (Reynolds et al., 2015). Previous studies have demonstrated that the overexpression of MCFA specific TE such as bacterial *TesA'* or plant *Umbellularia californica FatB2* (*UcFatB2*) alleviates the feedback inhibition caused by acyl-ACPs and yields MCFAs (Wang et al., 2012; Cao et al., 2016). Compared to *CnFatB1, CnFatB2* from *Cocos nucifera* and *UcFatB2*, the expression of *CnFatB3* in *Nicotiana benthamiana* results in the highest MCFA (Yuan et al., 1995; Reynolds et al., 2015). However, *CnFatB3* was not evaluated in *E. coli* in those studies. CcFatB1 from *Cinnamomum camphora* shows high specificity mainly for C14:0 in *E. coli* and *N. benthamiana* leaves (Yuan et al., 1995; Reynolds et al., 2015). CcFatB1 is also used for production of MCFA ethyl esters (Fan et al., 2013). CpFatB1 from *Cuphea palustris* with preference for C8 has been expressed in yeast for synthesis of short chain fatty acids, while CpFatB2 exhibits the highest specificity for C14:0 (24 mol%) when expressed in *Camelina* seeds (Kim and Chen, 2015). Bacterial LpFat from *Lachnoclostridium phytofermentans* shows preference for both C12 and C14 substrates, while CtFat from *Clostridium tetani* utilizes mainly C14 as substrate (Nawabi et al., 2011). Therefore, application of MCFA-specific TEs is crucial for the production of desired MCFAs and their derived HFAs.

In this study, three plant TE genes, *CnFatB3, CcFatB1*, and *CpFatB2* from *C. nucifera C. camphora*, and *C. palustris*, respectively, as well as the two bacterial TE genes, *CtFat* and *LpFat*, were first assessed in *E. coli*. The selected TE-encoding gene was then coexpressed with *P450*_*BM*3_ to produce medium chain HFAs in *E. coli*. Next, the effect of a fatty acid metabolism regulator *fadR* and an acyl-CoA synthase encoding gene *fadD* on HFA production was investigated. In addition, we also tested if supplementation of renewable glycerol could further improve the biomass as well as the yield of medium chain HFAs.

## Materials and methods

### Materials

Restriction enzymes, polymerase, and ligase were purchased from New England Biolabs (Beverly, USA). The plasmid miniprep kit, total DNA extraction kit, PCR purification kit, and gel extraction kit were purchased from TianGen (Beijing, China). Primers and genes were synthesized by TsingKe (Beijing, China). Fatty acid standards were purchased from Larodan (Stockholm, Sweden). Chemical reagents were purchased from Sigma-Aldrich (St. Louis, USA) or Sinopharm (Shanghai, China). His-tag and S-tag antibodies were purchased from Proteintech (Wuhan, China).

### Microbial strains and plasmids

Bacterial strains and plasmids were listed in Table S1. *E. coli* DH5α was used for plasmid construction and propagation. *E. coli* BL21(DE3), *E. coli* Rossetta(DE3), or *E. coli* BL21Δ*fadD* were used for FFA and HFA production. Primers used for plasmid construction are summarized in Table S2.

Plant TE genes *CnFatB3, CcFatB1, CpFatB2*, and bacterial TE genes *CtFat, LpFat*, based on GenBank accession numbers JF338905, U31813, U38189, CTC_RS00430, and ABX40638 respectively, were chemically synthesized with codon optimized for *E. coli*. The gene *P450*_*BM*3_ (GenBank accession AAA87602) from *B. megaterium* ATCC 14581 responsible for the ω-HFA synthesis was chemically synthesized.

For overexpression of individual TE genes, each gene was inserted into the *Eco*RI/*Xho*I double-digested pET28a(+). For co-expression of TE gene and *P450*_*BM*3_, *P450*_*BM*3_ was inserted into *Eco*RI/*Not*I sites and the TE gene was inserted into *Bgl*II/*Xho*I sites of the pCDFDuet-1 or pACYCDuet-1, respectively.

Fatty acid metabolism regulator FadR (NP_415705) from *E. coli* MG 1655, and the putative fatty acid metabolism regulators predicted based on *Rhodococcus opacus* PD630 genome sequence namely RoTetR1 (AHK36109), RoTetR2 (AHK34097), and RoTetR3 (AHK34039), were used in this study. The gene *fadR* was amplified with primers *fadR*-F and *fadR*-R (Table S2). The PCR product was inserted into the *Bam*HI and *Hind*III sites of pET28a(+), resulting in plasmid pEF. *R. opacus* RoTetR1/2/3 were chemically synthesized, and inserted into the *Bam*HI and *Hind*III sites of pET28a(+), resulting in plasmid pER1, pER2, or pER3, respectively.

### Detection of recombinant proteins by SDS-PAGE and western blot analysis

*E. coli* cells were cultivated at 37°C in Luria-Bertani medium (LB medium, 5 g/L yeast extract, 10 g/L tryptone, and 10 g/L NaCl) supplemented with corresponding antibiotics until their OD_600_ reached 0.6. Then, cells were cultured at 30°C for 4 h after addition of 1 mM isopropyl ß-D-thiogalactoside (IPTG). Cells were subsequently collected from 1.5 mL of bacterial cultures by centrifugation and re-suspended in sodium dodecyl sulfate (SDS) sample buffer. Proteins were heated in boiling water for 5 min. The resulting supernatant was analyzed by SDS-polyacrylamide gel electrophoresis and Western blot.

For Western blot analysis, protein samples were separated on a 12% polyacrylamide gel and transferred to a nitrocellulose membrane. After blocking non-specific binding using 5% skim milk for 1 h, the blots were incubated with anti His-tag monoclonal antibody or anti S-tag antibody at 4°C overnight. Membranes were washed three times with TTBS buffer (20 mM Tris-HCl, 150 mM NaCl, 0.1% Tween 20, pH 7.6) and incubated with an appropriate secondary antibodies conjugated to horseradish peroxidase for 1 h at room temperature. After being washed three times with TTBS, direct chemiluminescence imaging of the blots was performed using the ChemiDox XRS (BioRad) imaging system.

### Culture conditions for fatty acid production

For HFA production, cells were cultivated in flasks in M9 medium (15.13 g/L Na_2_HPO_4_·12H_2_O, 3 g/L KH_2_PO_4_, 1 g/L NH_4_Cl, 0.5 g/L NaCl, 2 mM MgSO_4_) containing 2% (w/w) glucose at 37°C with shaking at 200 rpm. Corresponding antibiotics were added when necessary. When the optical density of the cells reached 0.6 at 600 nm, IPTG was added to a final concentration of 1 mM. The cultures were incubated at 30°C with shaking at 150 rpm for further 16 h. Glycerol at final concentrations of 0, 0.5, 1, 2, 3, 4, and 5% were added into M9 medium with 2% glucose before culturing when tested. Further optimization of glucose concentration was also performed at final concentrations of 0, 0.5, 1, 1.5, and 2% in M9 medium containing 2% glycerol.

### Extraction of fatty acid from cell lysate and supernatant

The cell pellet and the supernatant of the cultures were collected separately. The cells were freeze-dried overnight while the supernatants were used to extract fatty acids directly. Before extraction, C21:0-FFA (25 μg) and 3-OH-C16:0-FFA (25 μg) were added to the supernatant as internal standards, respectively. Four milliliters of supernatants were first acidified with acetic acid. The total lipids were extracted with 2 mL of ethyl acetate by vigorous vortexing for 30 min. The solvent in the collected ethyl acetate layer was blown off with nitrogen and the lipids were methylated with 1 M HCl/CH_3_OH at 80°C for 3 h to generate fatty acid methyl esters (FAMEs). The FAMEs were then extracted with hexane. The dry cell pellets were methylated using the same methods to generate FAMEs. For detection of HFAs, the FAMEs were further converted into their trimethylsilyl (TMS) derivatives by incubating at 90°C for 1 h, with an excess of N,O-bis(trimethylsilyl) trifluoroacetamide (BSTFA).

### Fatty acid analysis

The FAMEs were analyzed using a 7890A gas chromatography (GC, Agilent technologies, USA) equipped with a flame ionization detector (FID) and an HP-FFAP column (30 m × 250 μm i.d. × 0.25 μm thickness). The oven temperature was initially set at 150°C for 1 min, then increased by 5°C/min to 230°C, holding at that temperature for 5 min. The temperature of the inlet and detector were 260 and 280°C, respectively. Structure of ω-HFAs was further confirmed by GC-mass spectrometry (GC-MS) analysis using Agilent 5975C with Triple-Axis detector. Two microliters of the sample were injected by split mode (split ratio 20:1) and analyzed using a nonpolar capillary HP-5 column (5% phenyl methyl siloxane capillary 30 m × 320 μm i.d. × 0.25 μm thickness). The oven temperature was set at 50°C for 1 min, and then increased by 15°C/min to 250°C, holding at this temperature for 10 min. The temperature of the inlet was 250°C, while the temperature of the detector was 280°C.

### Lipid analysis

Total lipids were extracted from dry cell pellets (20 mg) with chloroform-methanol (2:1, v/v) (Rottig et al., 2015). Prior to lipid extraction, 50 μg of internal standard C21:0-FFA was added into each sample. The lower organic phase was extracted, evaporated, and resolved. For thin layer chromatography (TLC), a certain amount of each sample was loaded on silica gel plates (silica gel 60, 20 × 20 cm, EMD Chemicals, Germany) with a solvent system containing hexane-diethyl ether-acetic acid (70:30:1, v/v). Lipid classes were visualized by spraying with 0.01% primuline (Sigma-Aldrich) in 80% acetone. The spots corresponding to FFA standard were scraped into glass vials and methylated as above. The resulting FAMEs were analyzed by GC as above.

## Results and discussion

### Assessment of plant and bacterial TEs for MCFA production in *E. coli*

**Figure 1:**
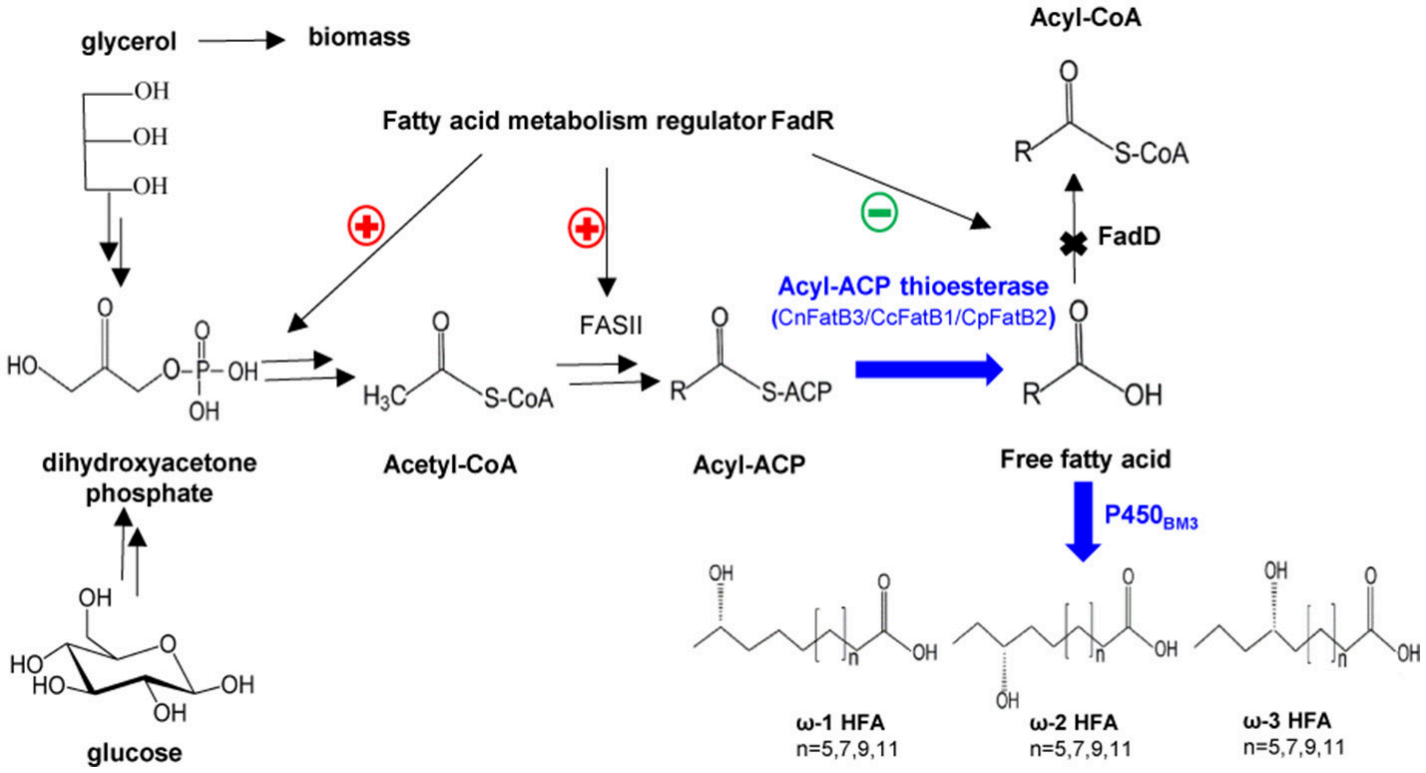
A schematic representation of the engineered pathway for production of medium chain ω-1/2/3 HFAs in *E. coli*. CnFatB3, CcFatB1, and CpFatB2 represent acyl-ACP thioesterases from *Cocos nucifera, Cinnamomum camphora*, and *Cuphea palustris*, respectively. FadD, acyl-CoA synthase from *E. coli*. P450_BM3_, P450 monooxygenase from *Bacillus megaterium*.

**Figure 2:**
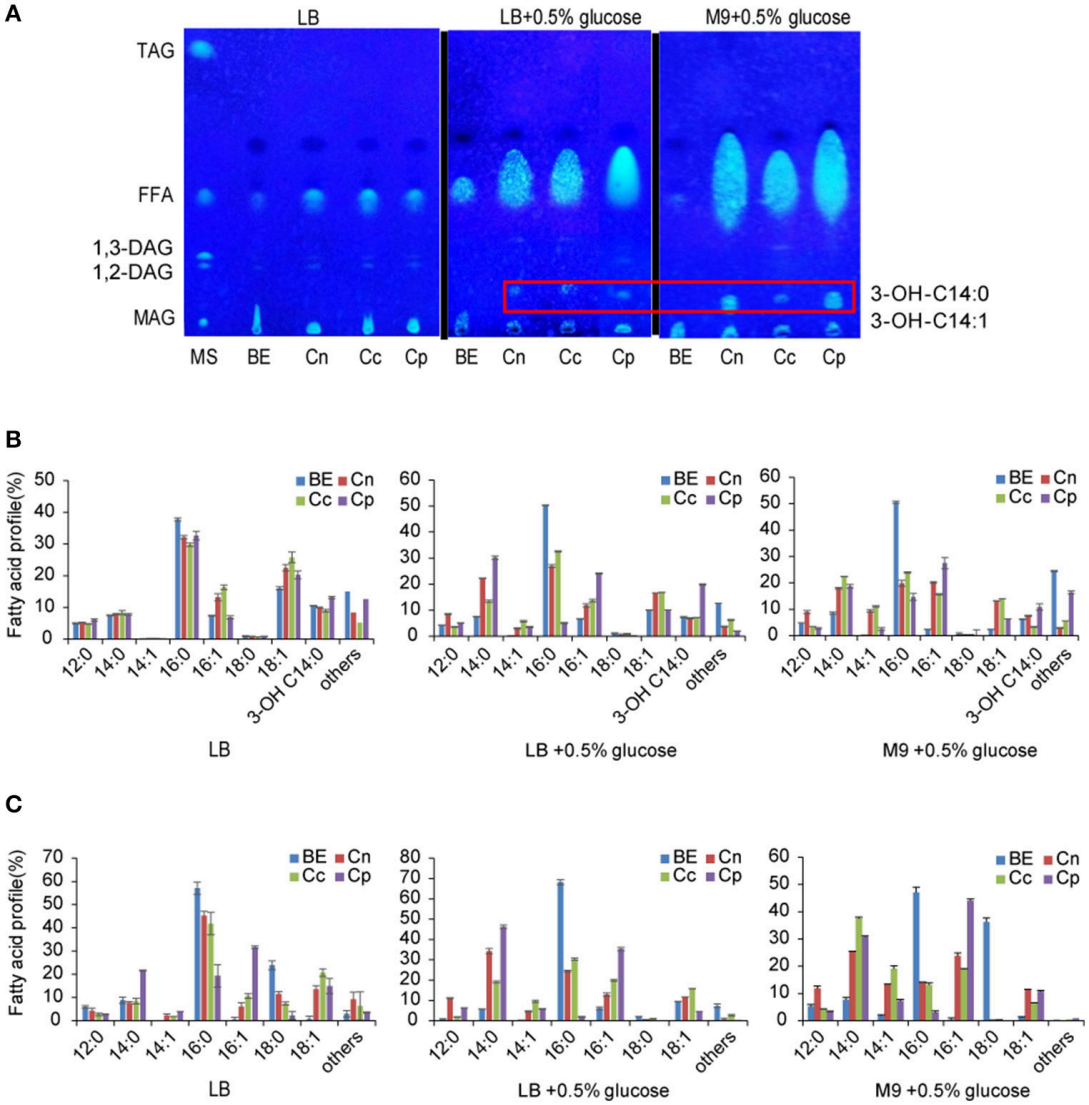
Overexpression of individual acyl-ACP thioesterase gene in *E. coli*. **(A)** Separation of neutral lipid from recombinant *E. coli* cell lysates by thin layer chromatography (TLC). **(B)** Fatty acid profile of cellular TFA of recombinant *E. coli*, trace amount of 3-OH-C14:1 was included in others. **(C)** Fatty acid profile of cellular FFA of recombinant *E. coli*. Trace amount of 3-OH-C14:0 and 3-OH-C14:1 were included in others. Cells were cultured in LB medium, LB medium plus 0.5% glucose, or M9 medium plus 0.5% glucose by IPTG induction for 24 h. MS, mixed standards for TLC containing 18:1/18:1/18:1-TAG, 18:1-FFA, 18:1/18:1-DAG, and 18:1-MAG; BE, BL21(DE3) with empty vector; Cn, BL21(DE3) harboring *CnFatB3* in pECn; Cc, BL21(DE3) haroboring *CcFatB1* in pECc; Cp, BL21(DE3) harboring *CpFatB2* in pECp. All data are the mean ± standard deviation from triplicate cultures.

For selective production of medium chain ω-1/2/3 HFAs, several steps involved in the biosynthesis pathway were tested in this study (Figure 1). The first step is to release MCFA as substrate for P450_BM3_ monooxygenase. Three plant TE genes, *CnFatB3, CcFatB1*, and *CpFatB2*, and two bacterial TE genes, *CtFat* and *LpFat*, were chosen to be expressed in *E. coli* BL21(DE3) individually. The neutral lipid fraction of total lipids from both cell pellet and supernatant were first separated by TLC. Only FFA and a small amount of diacylglycerol (DAG) were observed from cell neutral lipids after staining by primuline (Figure 2A), while in the supernatant there was only a FFA band observed (data not shown). Expressing TEs increased the cellular FFA amount, although there was no significant effect on the biomass and total fatty acid (TFA) amounts (Table 1). The amounts of MCFA including C12 and C14 had a slight increase in cellular FFA, but significantly increased in extracellular FFA, by expressing these TEs, compared to BL21(DE3) with empty vector BE (Table 1). The cellular content of C16:0 decreased while C14:0 and C14:1 increased, indicating that the expression of these TEs enhanced the release of MCFAs (Figures 2B,C, left panel, detailed profile shown in Tables S3, S4). Furthermore, CcFatB1 and CpFatB2 mainly released C14 FFA, while CnFatB3 released both C12 and C14 FFAs.

**Table 1:** A survey of acyl-ACP thioesterase activity on the production of TFA, FFA, and MCFA in recombinant *E. coli*.

**Medium/Strain**	**Biomass** **(mg)**	**TFA^a^** **(mg/L)**			**FFA^a^** **(mg/L)**			**C12 and C14** **(mg FFA/L)**			**C12 and C14** **(% cell or supernatant FFA)**	
		**Cell**	**Supernatant^b^**	**Total**	**Cell**	**Supernatant^b^**	**Total**	**Cell**	**Supernatant^b^**	**Total**	**Cell**	**Supernatant^b^**
**LB**												
BE^c^	44.6 ± 2.3	42.3 ± 4.4	22.4 ± 1.4	64.7	0.5 ± 0.1	22.4 ± 1.4	22.9	0.1 ± 0.0	0.6 ± 0.1	0.7	14.7 ± 1.2	2.6 ± 0.1
Cn^c^	33.2 ± 2.0	34.9 ± 1.5	25.5 ± 1.7	60.4	0.9 ± 0.0	25.5 ± 1.7	26.4	0.1 ± 0.0	5.2 ± 0.6	5.3	12.7 ± 2.3	20.2 ± 3.5
Cc^c^	47.1 ± 3.5	39.7 ± 5.2	24.3 ± 0.9	64.0	1.8 ± 0.2	24.3 ± 0.9	26.1	0.2 ± 0.1	5.0 ± 1.0	5.2	11.9 ± 1.7	20.8 ± 3.5
Cp^c^	43.3 ± 1.8	38.2 ± 2.9	19.9 ± 2.4	58.1	2.2 ± 0.1	19.9 ± 2.4	22.1	0.5 ± 0.1	3.7 ± 0.1	4.2	28.4 ± 1.7	18.5 ± 2.0
**LB+0.5% GLUCOSE**												
BE	84.5 ± 7.3	59.2 ± 10.9	8.9 ± 0.9	68.1	17.8 ± 2.8	8.9 ± 0.9	26.7	1.2 ± 0.2	0.3 ± 0.0	1.5	7.0 ± 0.1	3.5 ± 0.2
Cn	85.6 ± 3.7	114.0 ± 5.1	24.7 ± 2.5	138.7	72.4 ± 9.0	24.7 ± 2.5	97.1	36.0 ± 3.7	7.4 ± 1.3	42.4	49.8 ± 1.8	30.0 ± 2.0
Cc	95.6 ± 3.0	112.5 ± 6.6	16.0 ± 0.4	128.5	52.1 ± 4.4	16.0 ± 0.4	68.1	15.8 ± 1.6	1.7 ± 0.1	17.5	30.4 ± 1.2	10.8 ± 0.3
Cp	92.6 ± 2.1	194.4 ± 13.3	36.5 ± 1.2	230.9	156.3 ± 5.3	36.5 ± 1.2	192.8	91.2 ± 3.2	12.7 ± 0.5	103.9	58.3 ± 0.4	34.7 ± 4.2
**M9+0.5% GLUCOSE**												
BE	40.7 ± 5.1	28.1 ± 1.4	10.3 ± 0.0	38.4	0.4 ± 0.0	10.3 ± 0.0	10.7	0.1 ± 0.0	0.4 ± 0.1	0.5	14.7 ± 2.0	3.7 ± 0.4
Cn	52.7 ± 1.2	161.7 ± 12.4	122.9 ± 3.1	284.6	93.0 ± 8.5	122.9 ± 3.1	215.9	46.9 ± 3.3	57.9 ± 5.3	104.8	50.4 ± 1.2	34.5 ± 0.3
Cc	40.7 ± 5.7	93.0 ± 12.6	27.8 ± 0.4	120.8	47.3 ± 9.6	27.8 ± 0.4	75.1	28.8 ± 6.2	11.6 ± 0.7	40.4	60.9 ± 1.6	22.5 ± 0.7
Cp	39.5 ± 0.1	103.5 ± 7.8	86.0 ± 2.5	189.5	83.2 ± 4.6	86.0 ± 2.5	169.2	34.0 ± 5.0	23.4 ± 0.9	57.4	40.9 ± 3.1	20.7 ± 0.8
Ct^c^	55.8 ± 3.1	37.3 ± 1.7	12.5 ± 0.1	49.8	3.8 ± 0.4	12.5 ± 0.1	16.3	1.5 ± 0.4	3.7 ± 0.2	5.2	43.7 ± 5.8	39.5 ± 0.2
Lp^c^	34.9 ± 0.4	31.8 ± 3.7	11.3 ± 0.1	43.1	6.6 ± 0.4	11.3 ± 0.1	17.9	4.9 ± 0.4	3.6 ± 0.1	8.5	35.1 ± 0.4	74.2 ± 0.1

a *TFA, total fatty acids; FFA, free fatty acids; MCFA, medium-chain fatty acids*.

b *TLC separation of supernatant total lipid only detected a single FFA band, thus the amount of FFA from supernatant is calculated to be equal to TFA from supernatant*.

c *BE: BL21(DE3); Cn: BL21(DE3) harboring CnFatB3; Cc: BL21(DE3) harboring CcFatB1; Cp: BL21(DE3) harboring CpFatB2; Ct: BL21(DE3) harboring CtFat; Lp: BL21(DE3) harboring LpFat*.

Next, cell culture was supplemented with glucose in media. Supplementing 0.5% of glucose in LB medium significantly enhanced both biomass and TFA production compared to without glucose supplementation (Table 1). Importantly, all the recombinant cells expressing TEs had significantly higher TFA or FFA compared to BL21(DE3) expressing empty vector BE (Table 1), especially a huge increase in FFA (Figure 2A). Of them, strain Cp (*E. coli* harboring *CpFatB2*) yielded the highest TFA (194.4 mg/L), FFA (156.3 mg/L), and MCFA (91.2 mg/L) in the cell. Supplementing the same amount of glucose in M9 medium also increased the TFA but did not affect the biomass (Table 1). The TFA in the supernatant (appearing as FFA) were also increased in the presence of glucose except for the bacterial TEs. The ratio of FFA to TFA was about 60, 50, and 80% by Cn (*E. coli* harboring *CnFatB3*), Cc (*E. coli* harboring *CcFatB1*), and Cp, respectively, in both LB and M9 media with glucose (Table 1). Glucose could be converted into acetyl-CoA in *E. coli*, thus used for fatty acid synthesis. Indeed, TFA was enhanced in both LB and M9 media with supplementation of glucose. The increased biomass in LB medium but not M9 medium in the presence of glucose was probably due to the insufficient nitrogen in M9 medium.

HFAs in the form of FFA from cell lysate were also detected and later confirmed as the mixture of 3-OH-C14:0 and a trace amount of 3-OH-C14:1 by GC-MS (Figure S1). The 3-OH-C14:0 and 3-OH-C14:1 in FFA were visible on TLC plate (Figure 2A). It has been reported that the expression of TEs from *Clostridium acetobutylicum* and *C. phytofermentans* leads to a significant production of 3-OH-FFA with chain length ranging from C10 to C14 (Nawabi et al., 2011). However, the authors did not observe the same phenomenon when *Arabidopsis thaliana TE* (*AtFatA*) was overexpressed in *E. coli*, and thus it was concluded that plant TE could not lead to the production of 3-OH-FFA (Nawabi et al., 2011). In this study, the TLC result clearly demonstrated that plant TEs, CnFatB3, CcFatB1, and CpFatB2, led to the formation of 3-OH-FFA in *E. coli* as well. The native enzyme responsible for the formation of such HFA in *E. coli* has not been resolved yet.

Reduced C16:0 and increased C14:0, C14:1, or C16:1 caused by expressing plant TE was further enhanced by supplementation of glucose. C16:0 proportion in cellular TFA was sharply decreased from about 50% in the cells carrying empty vector (BE) to less than 20% in all the recombinant cells when supplied with glucose in either LB or M9 medium (Figure 2B). A similar effect was also observed in cellular FFA (Figure 2C). The most dramatic change was found with C16:0 in FFA, decreased from 68% in BE to 2% in the cells expressing Cp, while C14:0 and C14:1 increased from about 6% in BE to 50% in Cp in LB media with glucose (Figure 2C). Glucose could be converted to acetyl-CoA via glycolysis in *E. coli*, and subsequently condensed to yield acyl-ACP through the FASII system, leading to the enhanced FA synthesis.

CnFatB3 produced the highest TFA (284.6 mg/L), FFA (215.9 mg/L), and MCFA (104.8 mg/L) when cultured in M9 plus 0.5% of glucose, whereas CpFatB2 yielded the highest TFA (230.9 mg/L), FFA (192.8 mg/L), and MCFA (103.9 mg/L) in LB plus 0.5% of glucose. Unfortunately, two bacterial TEs generated much less FFA and MCFA contents than those released by plant TEs, although the proportion of C12 and C14 to the FFA was elevated as well (Table 1). Thus three plant TEs were selected for subsequent experiments.

### Sequence comparison of MCFA-specific TEs

The primary amino acid sequences and tertiary structures of these TEs were also compared. Amino acid sequence alignment showed that CnFatB3, CcFatB1, and CpFatB2 shared 74.2, 74.4, and 42.0% identities to UcFatB2, respectively. All these four TE proteins had the conserved residues NXHX_34_C as indicated in Figure S2A. Only CnFatB3 and CcFatB1 had the putative plastid-targeting peptide cleavage site (LPDW) which was located at the N-terminus (Figure S2A), as predicted for many other plant TEs. N-terminal domain is responsible for the enzyme specificity of plant TEs. Although the sequences of two bacterial TEs were much shorter than those of plant TEs, they also had conserved NXH structure. This might partly explain the much lower TFA, FFA, and MCFA contents obtained from bacterial TEs compared with those from plant TEs. No transmembrane structure was predicted among all these enzymes by the TMHMN tool (http://www.cbs.dtu.dk/services/TMHMM/). 3D models of CnFatB3, CcFatB1, and CpFatB2 were achieved through online software (http://zhanglab.ccmb.med.umich.edu/I-TASSER/, Figure S2B). The C-score of modeled CnFatB3, CcFatB1, and CpFatB2 were −2.75, −2.04, and −2.52, respectively, which were relatively low. They were all modeled based on the structure of oleoyl TE from *Lactobacillus plantarum* (PDB file: 2OWN), which shows preference for C6 and C8 acyl-ACP substrates. This is partly due to the lack of tertiary structural information on other TEs, especially medium chain acyl-ACP TEs. Nevertheless, a hot-dog fold pattern was found in CnFatB3, CcFatB1, and CpFatB2, which was described as the active site of the structure-resolved acyl-ACP TEs (PDB file: 2OWN and 2ESS). In addition, a few catalytic residues, for example Thr124 and Val183 in CcFatB1, Gly150, and Tyr234 in CpFatB2, were predicted. The above analysis of tested TEs might guide further improvement or application of those enzymes although the existing information on tertiary structures was limited.

### Increased ω-1/2/3 medium chain HFA content in *E. coli* by co-expression of *P450*_*BM*3_ and plant acyl-ACP thioesterase gene

**Figure 3:**
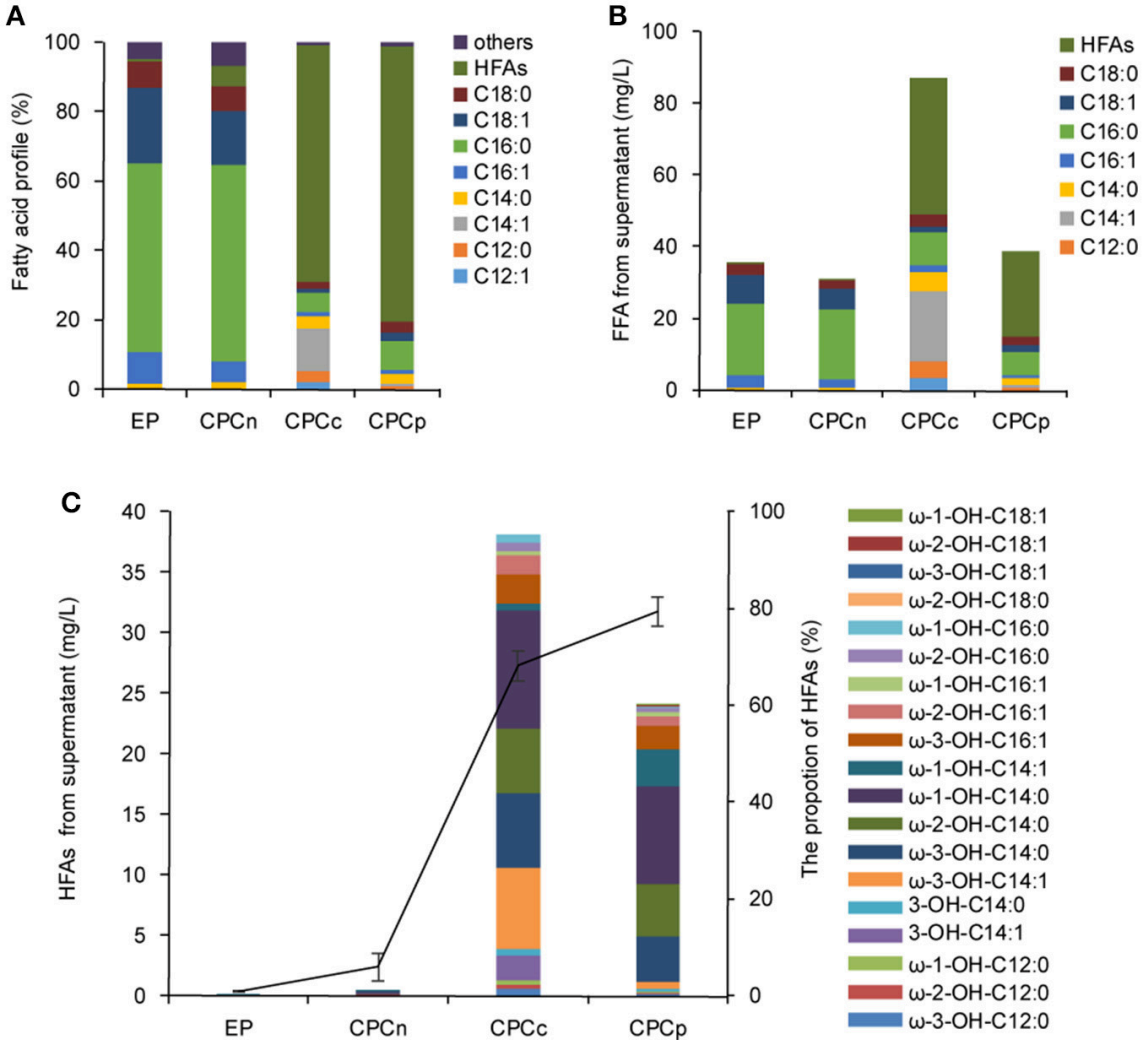
Coexpression of *P450*_*BM*3_ and plant thioesterase gene in *E. coli*. **(A)** Fatty acid profile of the supernatant FFA from the recombinant cells. **(B)** Fatty acid concentration of the supernatant FFA. **(C)** The concentration and the profile of the supernatant HFA. Black line represents the percentage of HFAs in the supernatant FFA. EP, BL21 harboring *P450*_*BM*3_ alone; CPCn, BL21 harboring *P450*_*BM*3_ and *CnFatB3*; CPCc, BL21 harboring *P450*_*BM*3_ and *CcFatB1*; CPCp, BL21 harboring *P450*_*BM*3_ and *CpFatB2*. All data are the mean ± standard deviation from triplicate cultures.

**Figure 4:**
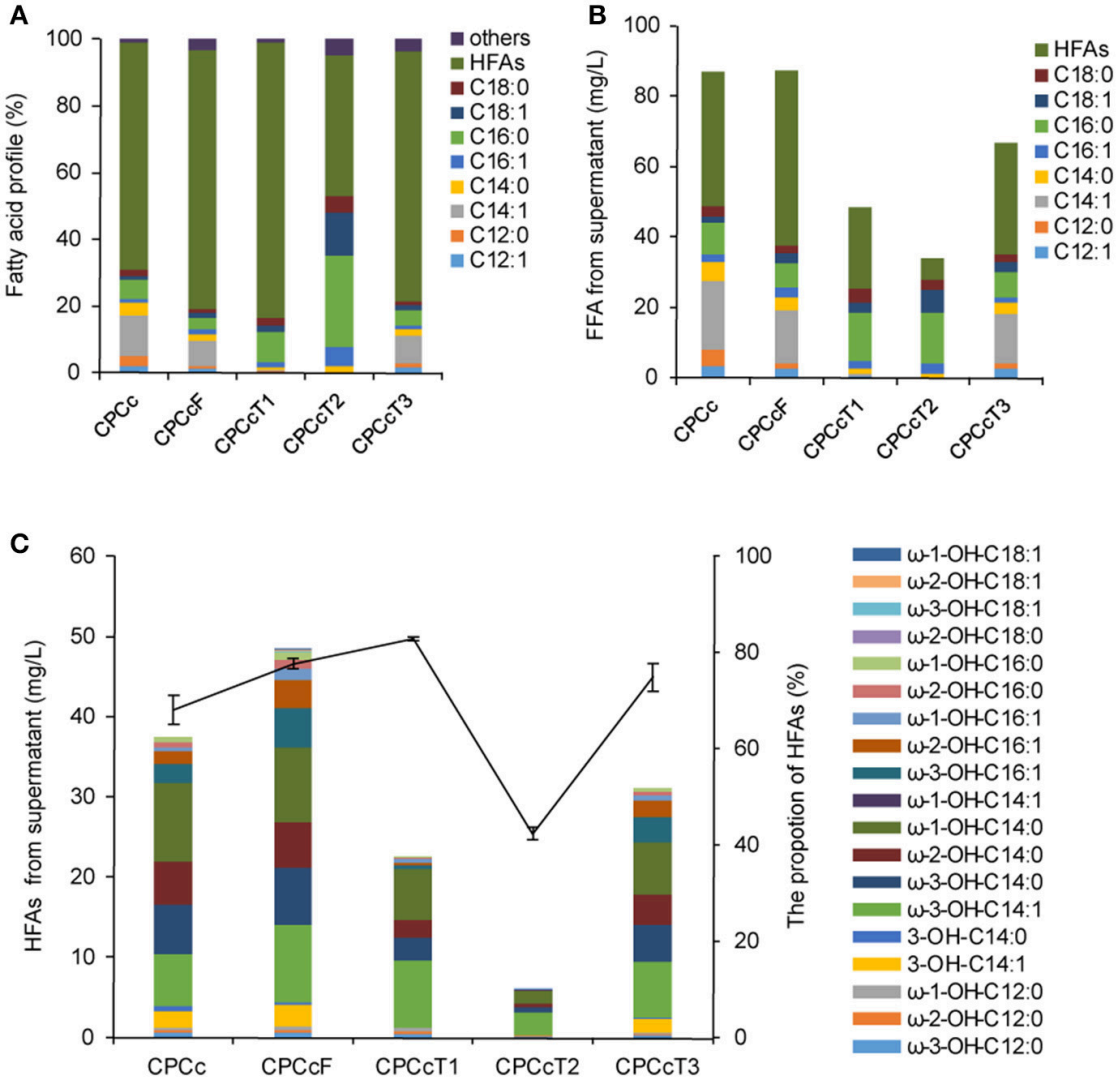
Coexpression of *P450*_*BM*3_, *CcFatB1* with additional fatty acid metabolism regulators in strain CPCc. **(A)** Fatty acid profile of the supernatant FFA. **(B)** The concentration of the supernatant FFA. **(C)** The concentration of HFAs from supernatant FFA. CPCc, BL21 harboring *P450*_*BM*3_ and *CcFatB1*; CPCcF, BL21 harboring *P450*_*BM*3_, *CcFatB1*, and *fadR* from *E. coli*; CPCcT1~CPCcT3, BL21 harboring *P450*_*BM*3_, *CcFatB1* and *RoTetR1*, or *RoTetR2*, or *RoTetR3* from *R. opacus* PD630. The vector pCDFDuet-1 is used for coexpression of *P450*_*BM*3_ and *CcFatB1*. pET28a is used for expression of individual *fadR, RoTetR1, RoTetR2*, or *RoTetR3*. All data are the mean ± standard deviation from triplicate cultures.

The sub-terminal carbons of released FFAs were then hydroxylated by a self-sufficient and region-selective P450_BM3_ to produce ω-1/2/3 HFAs. Such HFAs are valuable and more stable than terminal ω-HFAs. We first constructed the strains CPCn (*E. coli* harboring *P450*_*BM*3_ and *CnFatB3*), CPCc (*E. coli* harboring *P450*_*BM*3_ and *CcFatB1*), and CPCp (*E. coli* harboring *P450*_*BM*3_ and *CpFatB2*), to co-express *P450*_*BM*3_ and a single plant TE gene. Western blot analysis confirmed the expression of both genes in each recombinant strain (Figure S3). *E. coli* cells expressing *P450*_*BM*3_ alone (EP) did not produce detectable ω-1/2/3 HFAs (Figure 3). When coupled *P450*_*BM*3_ with different plant TE genes, the recombinant strains produced significant amounts of HFAs as shown in Figure 3. HFAs were comprised of 5.9, 68.1, and 79.4% of total FFA in the supernatant of CPCn, CPCc, and CPCp, respectively (Figure 4A). Among those combinations, strain CPCc yielded the highest amount of HFAs, which was up to 38.1 mg/L in the medium (Figures 3B,C). Unlike the previous results of single TE expression without *P450*_*BM*3_, only a small increase of MCFAs from the cell lysate was detected in CPCn (6.3 mg/L), CPCc (7.2 mg/L), and CPCp (7.4 mg/L), compared to 5.7 mg/L in control EP (Figure 3). This suggested that the MCFAs could be further converted into corresponding HFAs by P450_BM3_ monooxygenase. To our surprise, CPCn could only produce up to 1 mg/L HFAs. Neither BL21(DE3) nor BL21 *Rosseta*(DE3) harboring pCPCn produced HFA higher than 1 mg/L in the medium (Figure 3C and data not shown). In addition, FFA content from the CPCn cell lysate dramatically decreased, even to the level of the control cells. However, Western blot results confirmed the proteins were expressed although the protein levels were low (Figure S3). The combination of CnFatB3 and P450_BM3_ might negatively affect the fatty acid metabolism in a mechanism yet to be understood.

Based on these results, there was a positive correlation between FFA production obtained in the TE-only strains and HFA production in the derived strain containing both the respective TE and P450_BM3_. However, when FFA reached 90 mg/L or above, the production of HFA was reduced (Table 1 and Figure 3). Thus, precise modular control of the expression ratio of P450_BM3_ and MCFA-specific TE may be important to the production of high HFA levels.

Although dominated by C14 HFA, as many as 19 HFAs including ω-1-OH-C12/C14/C16/C18, ω-2-OH-C12/C14/C16/C18, ω-3-OH-C12/C14/C16/C18, 3-OH-C14:0, and 3-OH-C14:1 were detected from the supernatant FFA in stains CPCc and CPCp and confirmed by GC-MS (Figure 3C). The mass spectra of the main HFAs are shown in Figure S4. The HFA profile discovered here was more complicated than that of previous reports (Wang et al., 2012; Cao et al., 2016), where only four types of HFAs including 9-OH-C10, 11-OH-C12, 10-OH-C16, and 12-OH-C18, were detected in recombinant *E. coli* harboring *P450*_*BM*3_ and *tesA'* (Cao et al., 2016). Although the production of multiple HFAs might complicate the downstream purification process, variable ratios of different HFAs in the mixture from different gene combinations might also provide the unique polymer property from these HFAs.

For medium chain HFAs, CPCc exhibited strong preference for producing ω-1/2/3-OH-C14, which was up to 31.2 mg/L, corresponding to 81.8% of total HFA. Of them, ω-3-OH-C14:1, ω-3-OH-C14:0, ω-2-OH-C14:0, and ω-1-OH-C14:0 were the most abundant (Figure 3C and Figure S5). This result was consistent with the previous *in vitro* result that P450_BM3_ oxidized C14:0 very efficiently (Schneider et al., 1998), thus yielded abundant ω-1/2/3-OH-C14:0 in this study. Besides, a large amount of monounsaturated ω-3-OH-C14:1 was also produced. Only a small amount of ω-1/2/3-OH-C12:0 was detected which might be partly due to the insufficient supply of free C12:0 in strains APCc or CPCc. Nevertheless, our results showed that co-expressing CcFatB1 or CpFatB2 with P450_BM3_ greatly increased the production of medium chain ω-1/2/3-HFAs.

### Co-expression of transcriptional factor further boosted the production of medium chain HFA in BL21(DE3) and BL21Δ*fadD*

Fatty acid metabolism regulator FadR from *E. coli* plays dual roles in both repression of fatty acid degradation and activation of fatty acid biosynthesis (Zhang et al., 2012). Therefore, we were interested to test if FadR would enhance HFA production. In addition, we were also interested in screening more similar regulators from oleaginous bacteria. Three putative fatty acid metabolism regulators of oleaginous bacterium *R. opacus* PD630 were predicted in this work and designated as RoTetR1, RoTetR2, and RoTetR3, based on the published genome sequence of this strain. RoTetR1/2/3 showed high homology to other TetR family proteins, although phylogeny analysis indicated that *E. coli* FadR was substantially different from the TetR family (Figure S6). FadR overexpression in *E. coli* has resulted in increased TFA (Zhang et al., 2012). In this study, three putative *R. opacus* fatty acid metabolism regulators RoTetRs were individually overexpressed in *E. coli* BL21(DE3). However, no obvious increase of TFA or change of fatty acid profile was observed with RoTetRs (Figure S7). Then each *fadR* or *RoTetR* was expressed in HFA-producing strain CPCc. Expression of FadR resulted in the increase of HFAs from 38.1 to 49.8 mg/L (Figure 4). In contrast, RoTetRs reduced the content of total FFA (Figure 4B), leading to the lower amount of HFAs. However, the ratio of HFA in total extracellular FFA was increased by RoTetR1 or RoTetR3 (Figure 4A). It has been reported that the overexpression of *fadR* enhances TFA titer by 7.5-fold, and the increased proportion of unsaturated fatty acids, which has been explained by FadR-mediated activation of *fabA* and *fabB* (Zhang et al., 2012). It could partly explain why unsaturated ω-3-OH-C14:1 was up-regulated (Figure 4C and Figure S8). Unlike previous reports, TFA yield was barely elevated in this study which might be due to the subsequent conversion of FFA by P450_BM3_. Our results showed that RoTetR alone had no obvious effect on the amount of TFA, but reduced the supernatant FFA when expressed in the HFA producing strain, with an increased HFA ratio in the supernatant FFA by RoTetR1 or RoTetR3.

**Figure 5:**
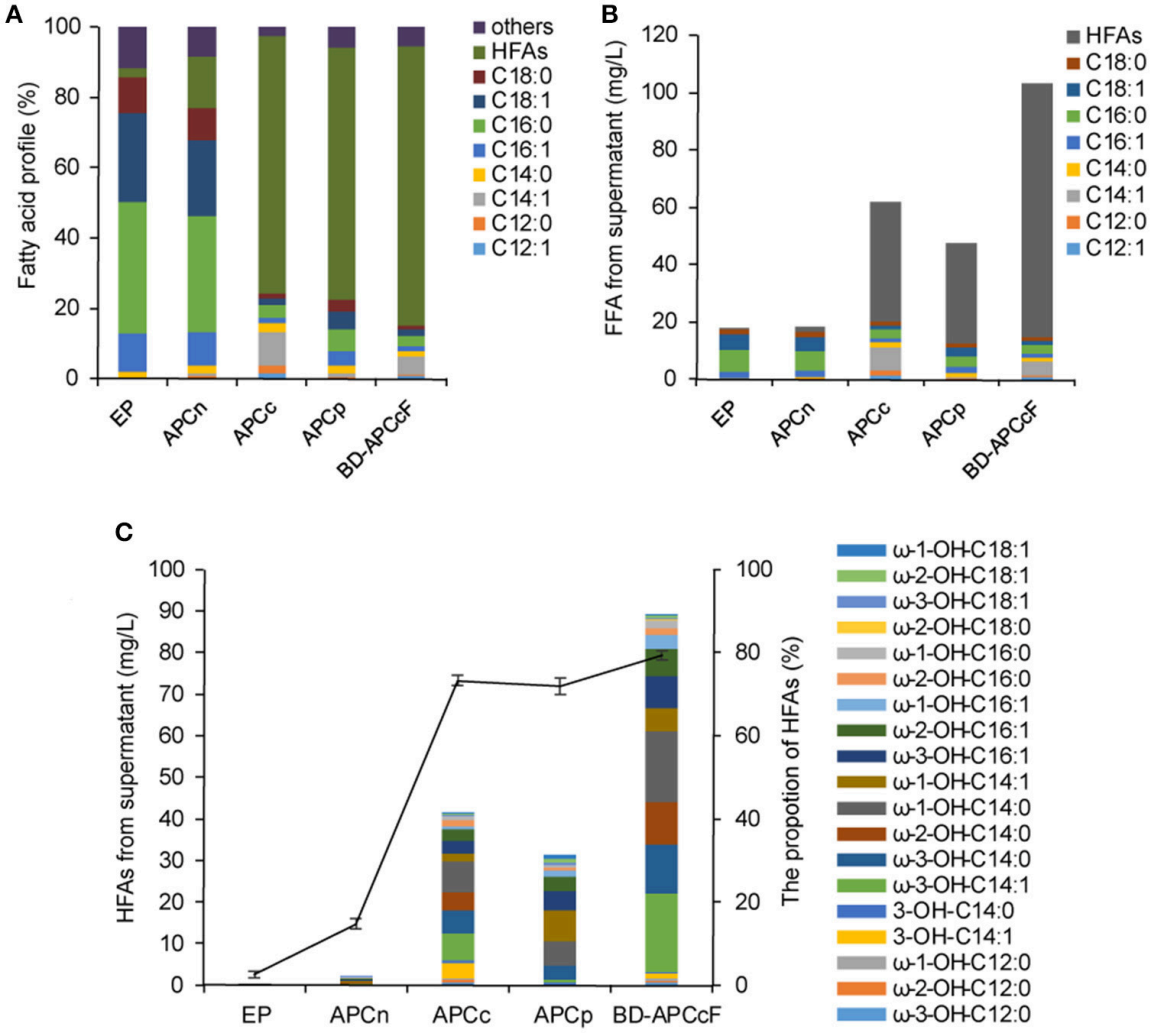
Coexpression of *P450*_*BM*3_, plant thioesterase gene and *fadR* in BL21 or BL21Δ*fadD*. **(A)** The proportion of the supernatant FFA. **(B)** The titer of each category of FFA. **(C)** The concentration of HFAs from the supernatant FFA. EP, BL21 harboring *P450*_*BM*3_ alone; APCn, BL21 harboring *P450*_*BM*3_ and *CnFatB3*; APCc, BL21 harboring *P450*_*BM*3_ and *CcFatB1*; APCp, BL21 harboring *P450*_*BM*3_ and *CpFatB2*, BD-APCcF: BL21Δ*fadD* harboring *P450*_*BM*3_, *CcFatB1*, and *fadR*. The vector used for *P450*_*BM*3_ and plant *TE* was pACYCDuet-1 instead of pCDFDuet-1 in this test. The cultures were incubated at 30°C with shaking at 150 rpm for 16 h after IPTG induction. All data are the mean ± standard deviation from triplicate cultures.

Using a low copy number vector for the expression of TE could alleviate the unintended consequences caused by modulating of acyl-ACP pools (Lennen and Pfleger, 2012). Thus pACYCDuet-1 with a copy number of about 10–12, which was much lower than that of pCDFDuet-1, was used in this study as well. Results showed that the production of HFAs by CcFatB1 from the low copy number vector in strain APCc was 41.8 mg/L, higher than 38.1 mg/L by CcFatB1 from the high copy number vector in strain CPCc (Figures 4C, 5C). Again, CnFatB3 from the low copy number vector in APCn only yielded 1.6 mg/L of HFAs (Figure 5C).

Inactivation of *fadD*, the only gene coding for acyl-CoA synthetase in *E. coli*, would substantially inhibit the degradation of fatty acids by preventing acyl-CoA synthesis (Fujita et al., 2007). Low copy number plasmid, pAPCcF, and high copy number plasmid, pCPCcF, were transformed in to BL21Δ*fadD* mutant respectively, for construction of *BD*-APCcF and *BD*-CPCcF. Indeed, HFAs concentration was doubled and reached up to 86.1 mg/L (Figures 5A,B). Notably, the levels of medium chain HFAs including ω-3-OH-C14:1, ω-3-OH-C14:0, ω-2-OH-C14:0, ω-1-OH-C14:0 were all doubled or tripled in *BD*-APCcF, up to 18.4, 11.5, 9.8, and 16.7 mg/L, respectively (Figure 5C and Figure S9). The concentration of ω-1/2/3-OH-C16:1 was enhanced as well, calculated as 17.2 mg/L, mainly due to the existence of naturally high amounts of C16:1 in *E. coli* (Figure S9). *BD*-APCcF produced higher amounts of HFA than *BD*-CPCcF, thus *BD*-APCcF was used in the following study. The utilization of pCDFDuet-1 led to a significantly lower amount of HFAs, when compared to pACYCDuet-1 in this study. They differed in the resistance marker (chloramphenicol vs. streptomycin), the type of replicon (CloDF13 vs. P15A) and the copy number (20–40 vs. 10–12). We thus proposed that a lower copy number vector might be beneficial for the high yield of HFAs in *E. coli*.

Since the translation efficiency decreased with the increasing distance from the gene to the promoter in expression plasmid, *CcFatB1* was then arranged after *P450*_*BM*3_ under the same T7 promoter in pCDFDuet-1 or pACYCDuet-1 (see Table S1, strains *BD-*APCc-1 and *BD-*CPCc-1). However, none of these constructs exceeded the HFAs production of *BD*-APCcF. The highest titer of HFAs produced by strain *BD-*APCc-1 achieved only 17.6 mg/L, while strain *BD-*CPCc-1 achieved 14.0 mg/L. Therefore, more precise fine-tuning of P450_BM3_ and CcFatB1 might be needed considering the balance of metabolism burden and product. Besides, β-oxidation reversal has been successfully applied to produce carboxylic acids and MCFAs (Clomburg et al., 2015; Wu et al., 2017). Theoretically, overexpression of genes responsible for β-oxidation reversal along with *P450*_*BM*3_ might further improve the desired HFA production by using acyl-CoA pool directly.

### Addition of glycerol led to higher HFA accumulation

**Figure 6:**
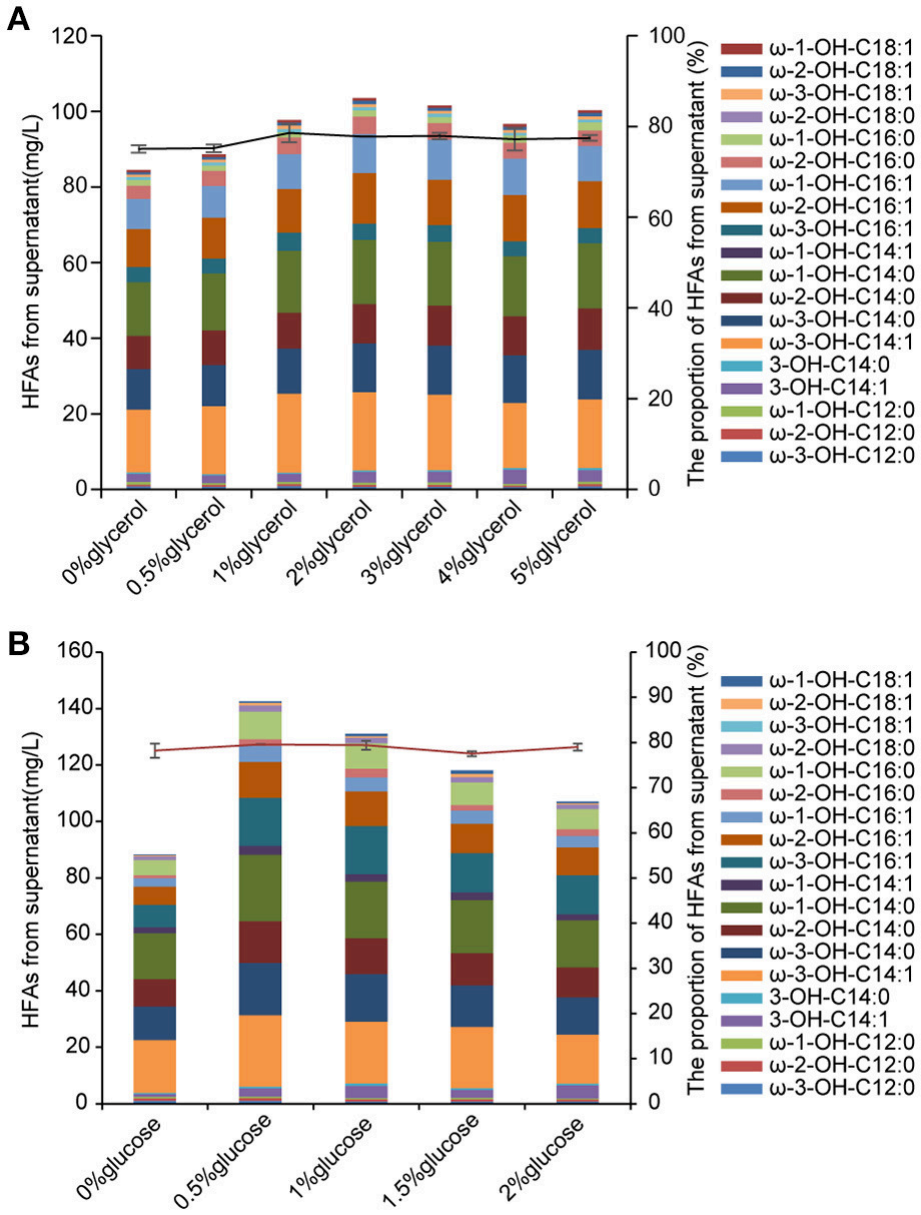
The effect of glycerol and glucose on HFAs production. **(A)** The concentration and the profile of HFAs from supernatant in M9 medium plus 2% glucose along with 0–5% glycerol. **(B)** The concentration and the profile of HFAs from supernatant in M9 medium plus 2% glycerol along with 0–2% glucose. All data are the mean ± standard deviation from triplicate cultures.

HFAs are intracellularly synthesized in *E. coli*, thus the biomass would be a key factor that determines the amount of produced HFAs. Glycerol as an inexpensive carbon source has been applied in cultivation of *E. coli* for enhanced biomass (Chen and Liu, 2016). In this study, 0.5–5% (w/v) of glycerol was added into the M9 medium containing 2% (w/v) glucose at the beginning of culturing. The biomass of *BD*-APCcF was indeed increased after incubating with different concentrations of glycerol, rising from 28 to 37 mg per 50 mL medium, although the biomass was still slightly lower than that of control EP, which was 39 mg. As expected, the HFA concentration of strain *BD-*APCcF was increased from 86.1 to 107.9 mg/L when supplemented with 2% of glycerol, although the HFA profile was not changed (Figure 6A).

In general, 2% of glucose was used for production of HFA in previously published work (Wang et al., 2012; Kim et al., 2015; Cao et al., 2016). Here, we found that the reduced amount of glucose to as low as 0.5% along with 2% glycerol in the medium led to 144 mg/L HFAs and 50.6 mg of cell dry weight. In addition, the HFA titer from the culture without supplementation of glucose was much lower than that of culture with 0.5–2% glucose (Figure 6B). The presence of glucose in medium could hinder *E. coli* to utilize other carbon resources such as lactose and glycerol due to the glucose effect. After consumption of glucose by cells for growth, glycerol could then be channeled into glycolysis via production of dihydroxyacetone phosphate, glyceraldehyde phosphate, and pyruvate. Pyruvate would subsequently be converted into acetyl-CoA, the precursor for fatty acid synthesis. Glycerol is cheaper than glucose, and more importantly, it has a higher degree of reduction than glucose, thus it is proposed that the theoretical yield of fatty acid, oils, or other reduced chemicals on glycerol is higher than that of glucose (Chen and Liu, 2016). With the combinational effect of glucose and glycerol, the HFA titer was further elevated. The cost of feed-stock accounts for a heavy proportion of the total cost of HFA production. Previous work that feeding glucose instead of FFA in culture is an important step forwards commercial application (Wang et al., 2012). Our results demonstrated that the addition of abundant, inexpensive and renewable glycerol with the reduction of glucose usage elevated the desired HFA production as well.

## Conclusion

This study demonstrated that the overexpression of plant acyl-ACP TEs from *C. nucifera, C. camphora*, or *C. plaustris* in *E. coli* substantially increased the production of MCFA both in cell and in supernatant. Co-expression of P450_BM3_, plant acyl-ACP TE, and fatty acid metabolism regulator FadR yielded substantially increased amount of ω-1/2/3 position HFAs, especially ω-1/2/3-OH-C14. Reducing usage of glucose and supplementing with cheaper glycerol further boosted the production of HFAs. Importantly, such HFAs were secreted into the medium which was convenient for downstream purification of HFAs. In summary, *E. coli* had been metabolic engineered to selectively produce medium chain omega HFA by utilization of renewable glucose and glycerol.

## Author contributions

KX, X-HY, LW, F-HH, and XW: conceived the study; KX, X-HY, and LX: conducted experiments; XW, KX, W-CC, and X-RZ: analyzed the data and wrote the manuscript; All authors read and approved the final manuscript.

### Conflict of interest statement

The authors declare that the research was conducted in the absence of any commercial or financial relationships that could be construed as a potential conflict of interest.

## Supplementary material

Figure S1 Mass spectra of 3-OH-C14:0 **(A)** and 3-OH-C14:1 **(B)** TMS derivatives. ^*^Indicates the characteristic mass ions.

Figure S2 Amino acid sequence comparison and structure prediction of Acyl-ACP TEs. **(A)** Alignment of amino acid sequences of CnFatB3 (JF338905) from *Cocos nucifera*, CcFatB1 (U31813) from *Cinnamomum camphora*, CpFatB2 (U38189) from *Cuphea plaustris*, UcFatB2 (U17097) from *Umbellularia californica, CtFat* (CTC_RS00430) from *Clostridium tetani* and *LpFat* (ABX40638) from *Lachnoclostridium phytofermentans*. The secondary structure elements of each protein are shown above the alignment. Identical amino acids are shown in white on a red background, while similar residues are shown in red. Three conserved residues that constitute the catalytic triad are indicated by black arrows. The start of mature proteins is indicated by blue arrow. **(B)** Structural prediction analysis of CnFatB3 (left), CcFatB1 (middle), and CpFatB2 (right).

Figure S3 Western blot analysis of recombinant cells. The proteins from cells harboring *P450*_*BM*3_ along with *CnFatB3* (left), *CcFatB1* (middle), or *CpFatB2* (right) were blotted with anti His-tag monoclonal antibody **(A)** for P450_BM3_ fusion protein or anti S-tag antibody **(B)** for TE fusion proteins.

Figure S4 The chemical structure and mass spectra of HFA TMS derivatives from strain CPCc. Mass spectra of 3-OH-C14:0 and 3-OH-C14:1 TMS derivatives were same as Figure S1. ^*^Indicates the characteristic mass ions.

Figure S5 Hydroxyl fatty acid profile for recombinant strain EP, CPCn, CPCc, and CPCp, respectively.

Figure S6 Phylogeny tree of three potential fatty acid metabolism regulators RoTetRs and FadR from *E. coli*.

Figure S7 Lipid profile of recombinant *E. coli* hells expressing individual *RoTetRs*. **(A)** TLC showing the neutral lipids; **(B)** fatty acid profile.

Figure S8 Hydroxyl fatty acid profile for recombinant strain CPCc, CPCcF, CPCcT1, CPCcT2, and CPCcT3, respectively.

Figure S9 Hydroxyl fatty acid profile for recombinant strain EP, APCn, APCc, APCp, and BD-APCcF, respectively.

Table S1 Strains and plasmids used in this study.

Table S2 Details of primer sequences used in this test.

Table S3 Fatty acid profile of cellular total fatty acid (TFA) produced by BL21(DE3) harboring individual plant acyl-ACP thioesterase gene.

Table S4 Fatty acid profile of cellular cell free fatty acid (FFA) a produced by BL21(DE3) harboring individual plant acyl-ACP thioesterase gene.

## Abbreviations

ACP: Acyl-carrier protein
TAG: triacylglycerol
DAG: Diacylglycerol
MAG: monoacylglycerol
FAME: Fatty acid methyl ester
FatB: Fatty acyl thioesterase B
FFA: Free fatty acid
HFA: hydroxyl fatty acid
IPTG: Isopropyl ß-D-thiogalactoside
MCFA: Medium chain fatty acid
SDS: Sodium dodecyl sulfate
TE: Acyl-ACP thioesterase
TFA: Total fatty acid
TLC: Thin layer chromatography
TMS: Trimethylsilyl.
