# Supplementary figures and images for: Metabolic Engineering for Enhanced Medium Chain Omega Hydroxy Fatty Acid Production in Escherichia coli
Source: Front Microbiol. 2018 Feb 7;9:139. doi: 10.3389/fmicb.2018.00139 (PMC5808347; doi:10.3389/fmicb.2018.00139)

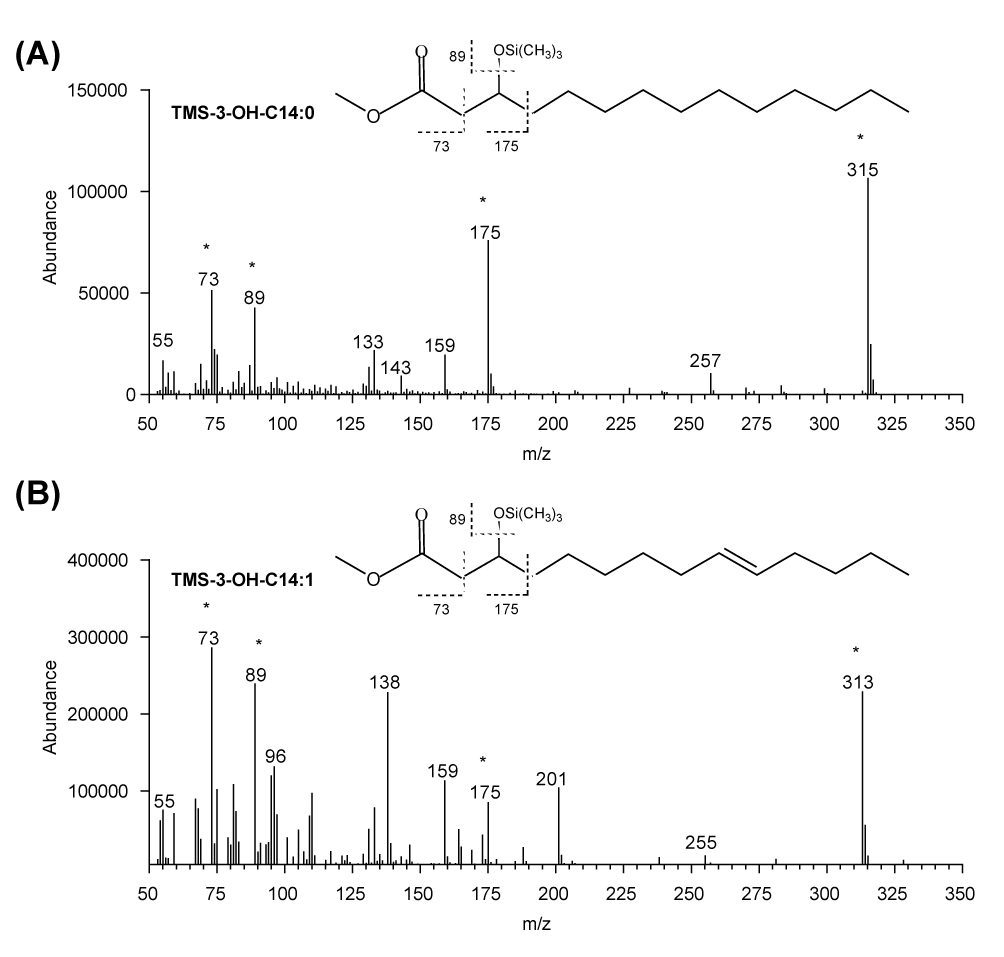

Supplement: Figure S1 — Mass spectra of 3-OH-C14:0 (A) and 3-OH-C14:1 (B) TMS derivatives. *Indicates the characteristic mass ions. [file Image1.TIF]

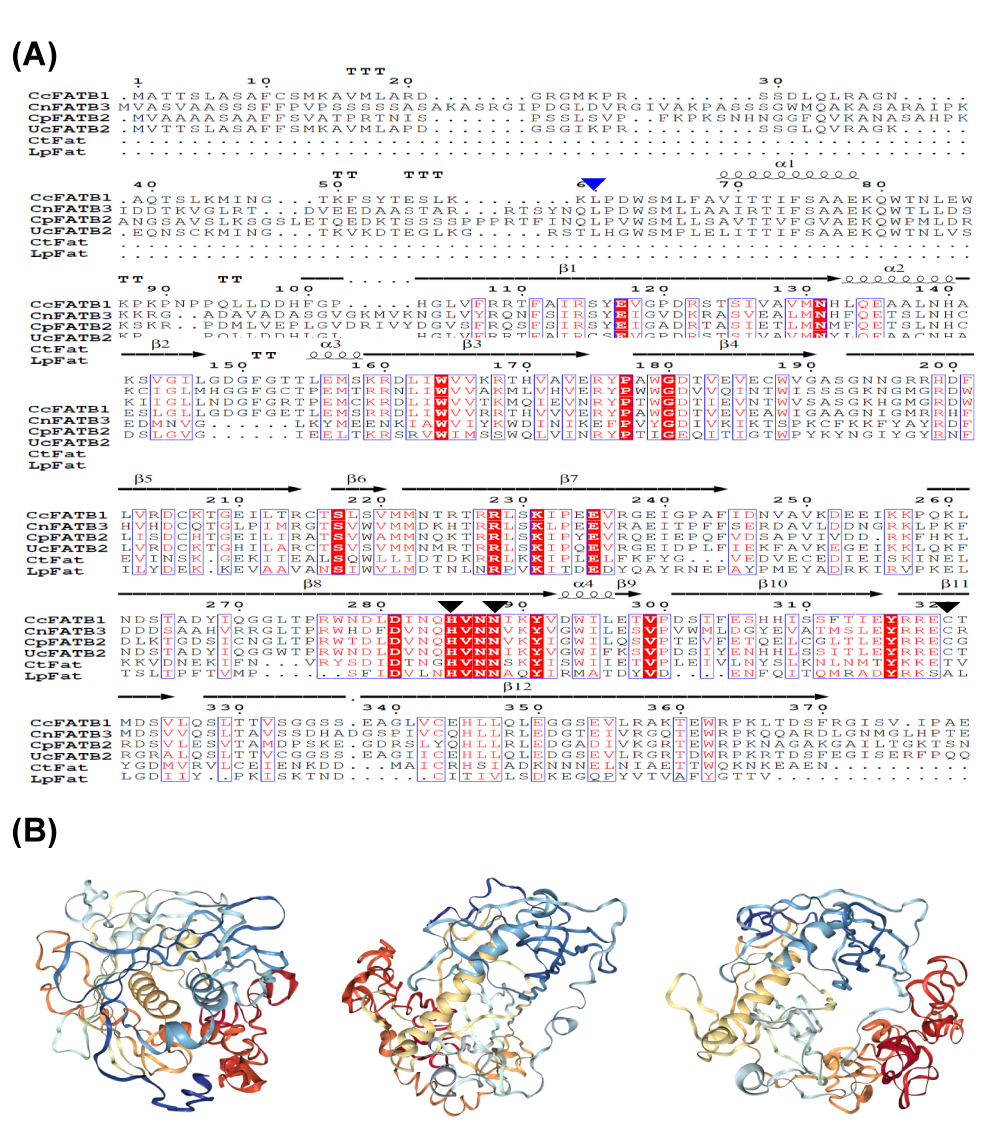

Supplement: Figure S2 — Amino acid sequence comparison and structure prediction of Acyl-ACP TEs. (A) Alignment of amino acid sequences of CnFatB3 (JF338905) from Cocos nucifera, CcFatB1 (U31813) from Cinnamomum camphora, CpFatB2 (U38189) from Cuphea plaustris, UcFatB2 (U17097) from Umbellularia californica, CtFat (CTC_RS00430) from Clostridium tetani and LpFat (ABX40638) from Lachnoclostridium phytofermentans. The secondary structure elements of each protein are shown above the alignment. Identical amino acids are shown in white on a red background, while similar residues are shown in red. Three conserved residues that constitute the catalytic triad are indicated by black arrows. The start of mature proteins is indicated by blue arrow. (B) Structural prediction analysis of CnFatB3 (left), CcFatB1 (middle), and CpFatB2 (right). [file Image2.TIF]

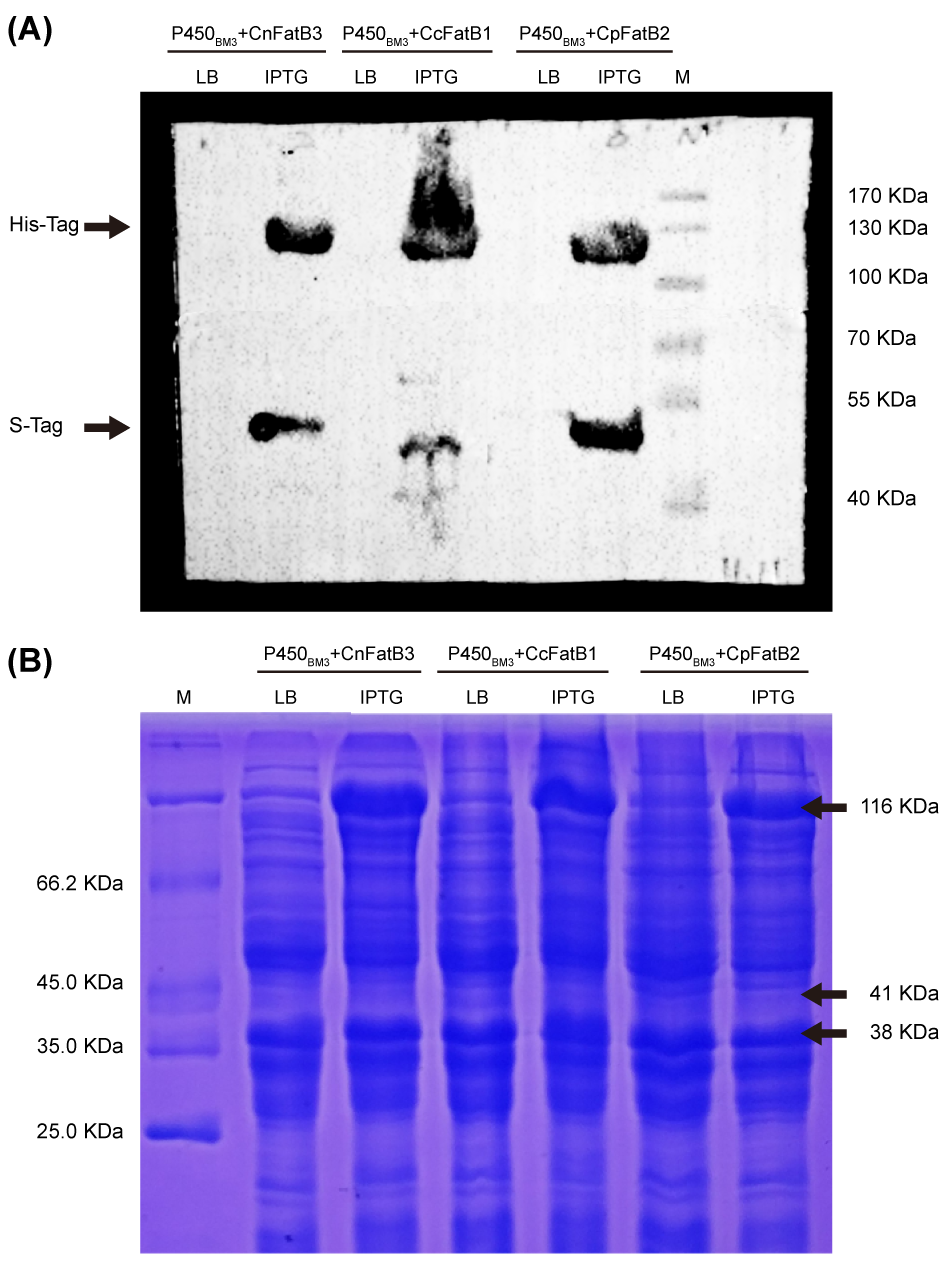

Supplement: Figure S3 — Western blot analysis of recombinant cells. The proteins from cells harboring P450BM3 along with CnFatB3 (left), CcFatB1 (middle), or CpFatB2 (right) were blotted with anti His-tag monoclonal antibody (A) for P450BM3 fusion protein or anti S-tag antibody (B) for TE fusion proteins. [file Image3.TIF]

**Figure S4** The chemical structure and mass spectra of HFA TMS derivatives from strain CPCc.

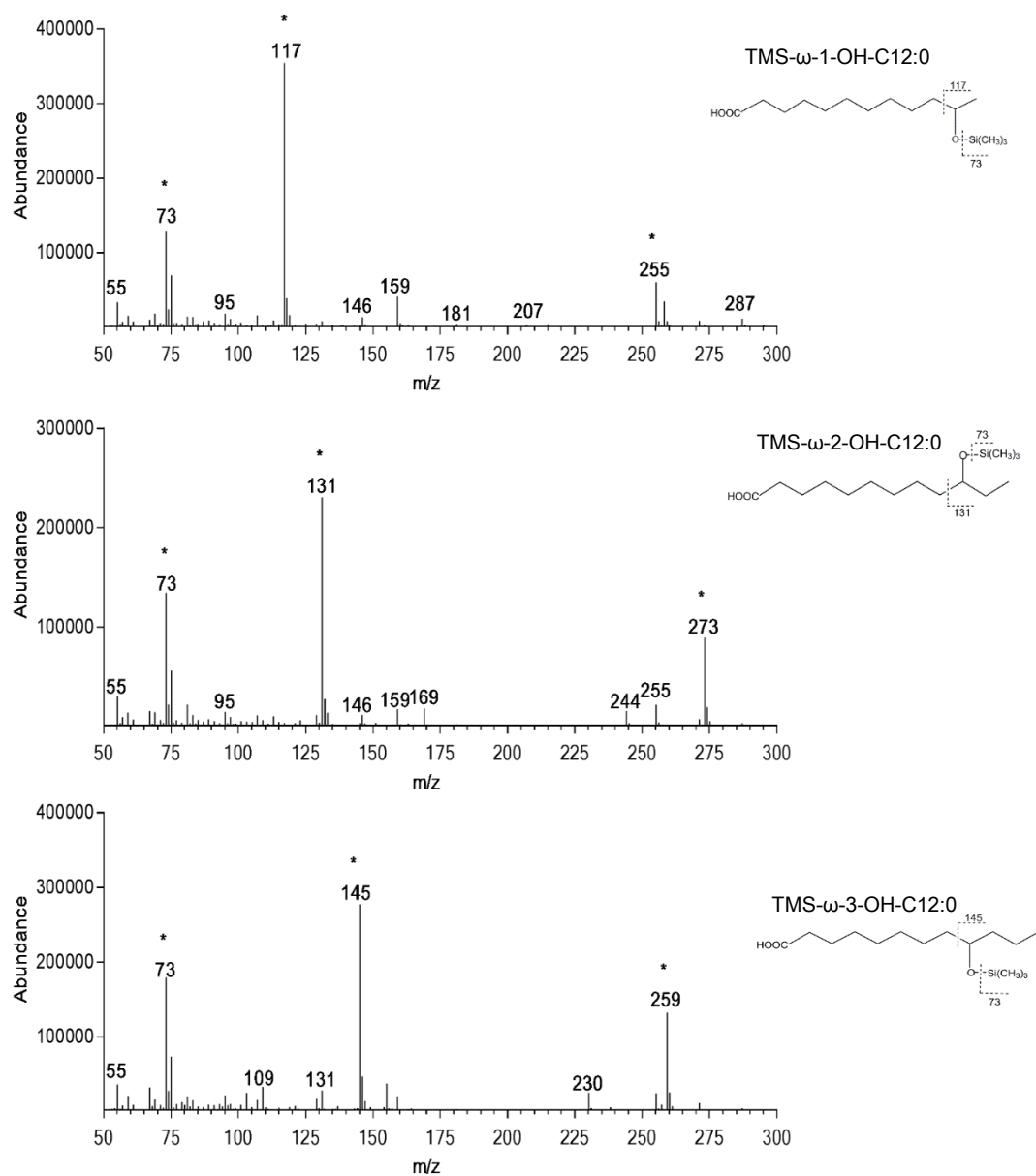

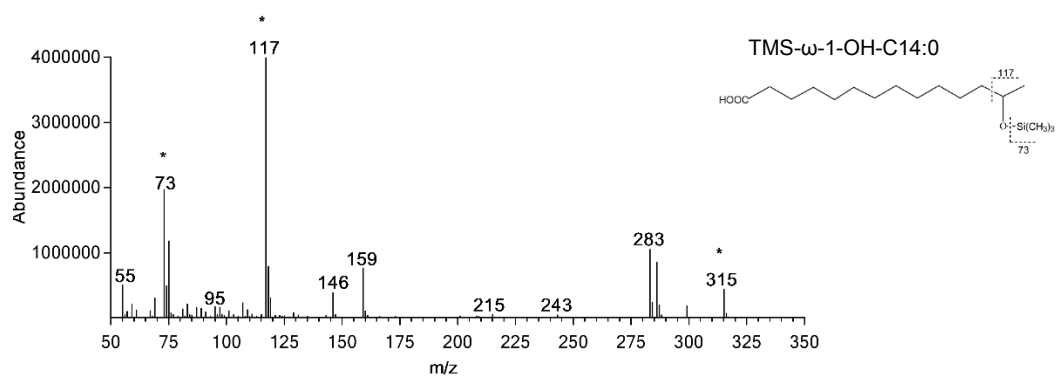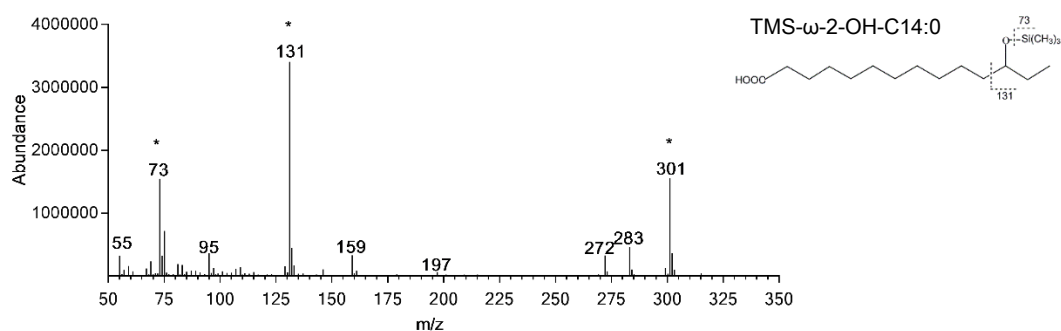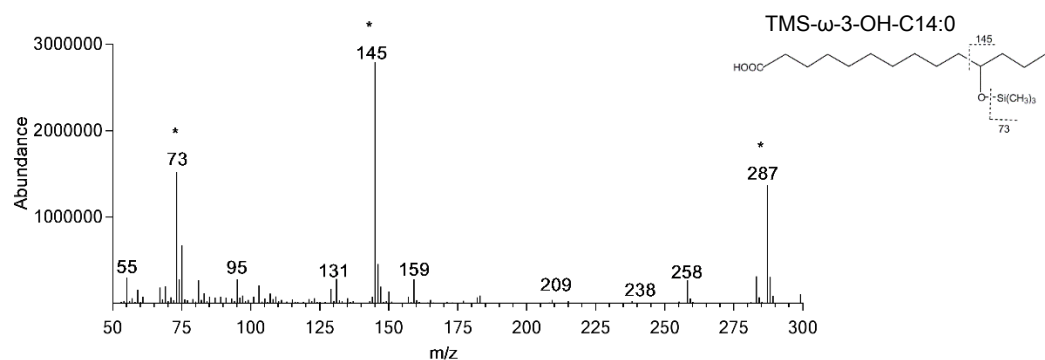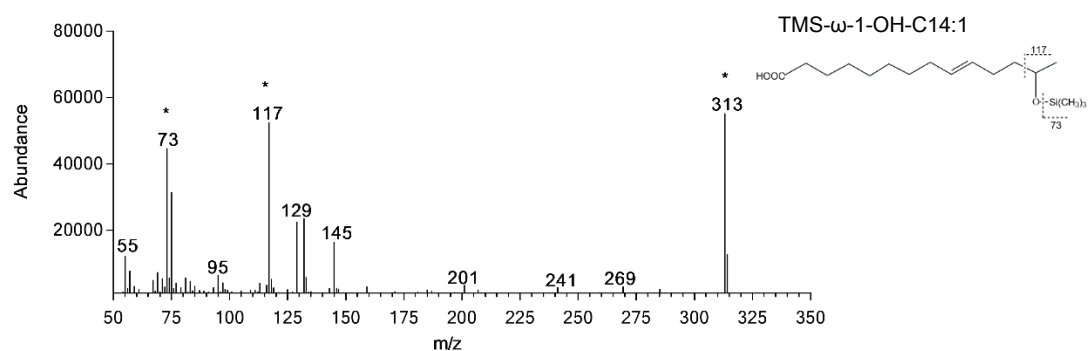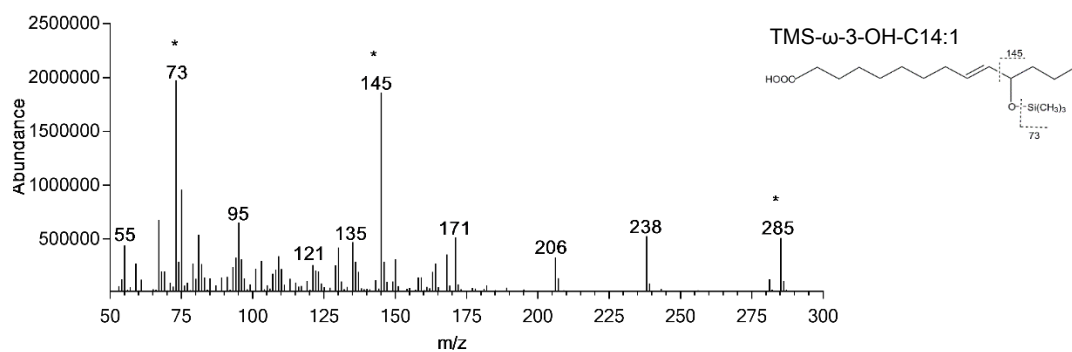

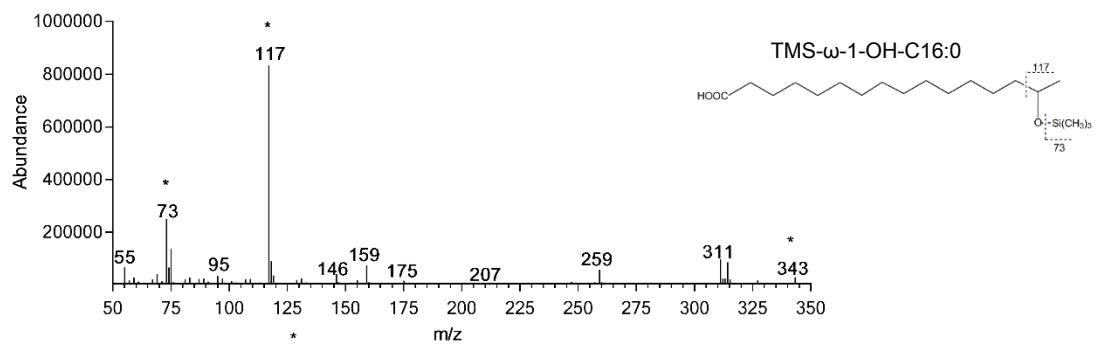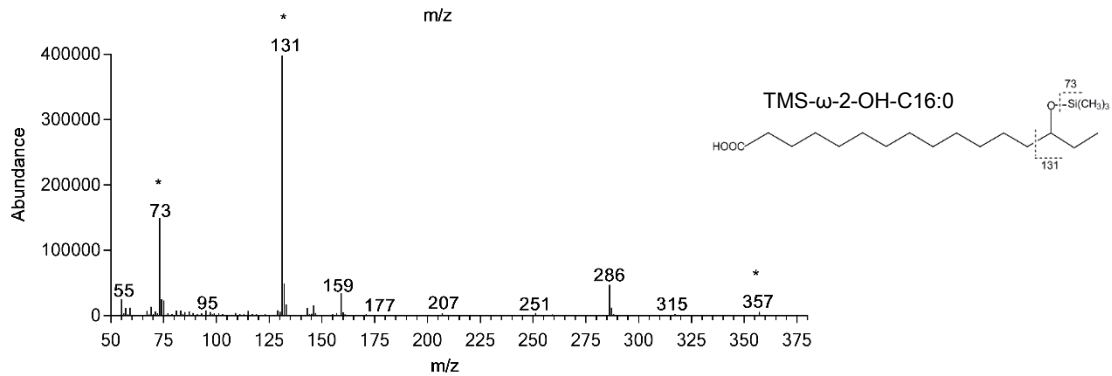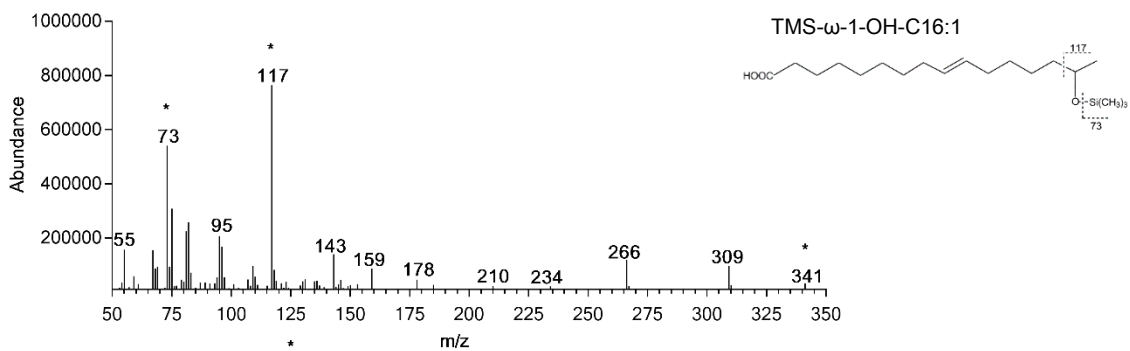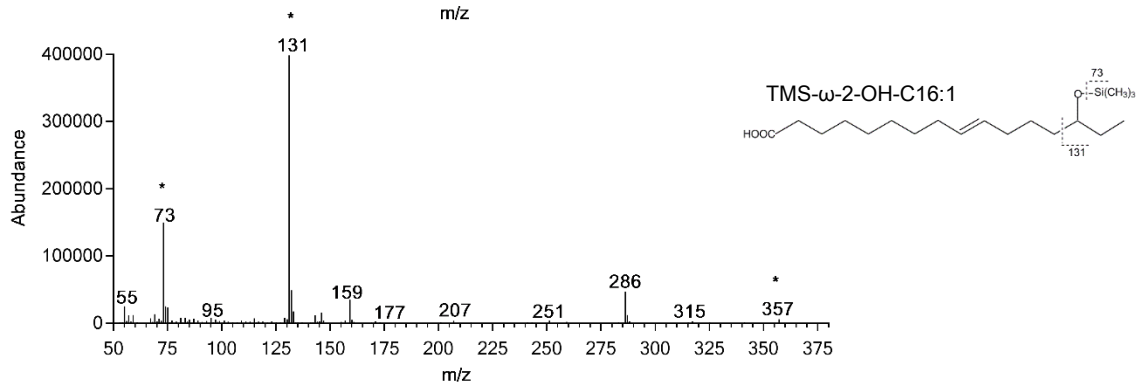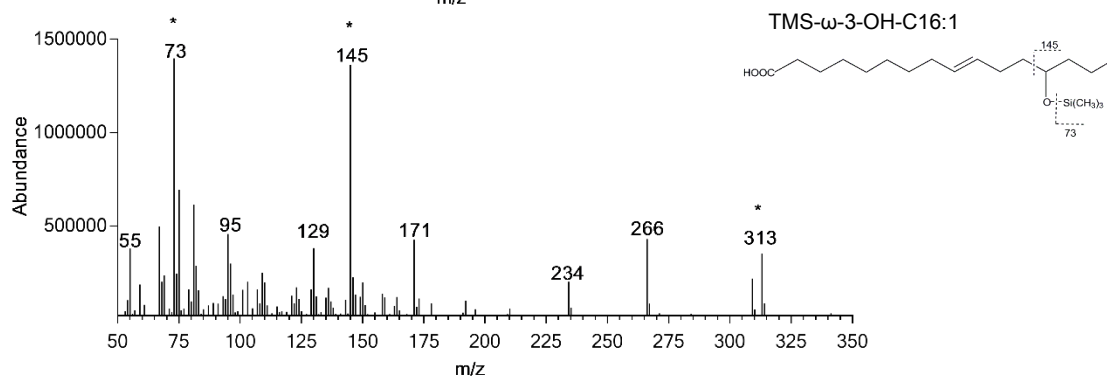

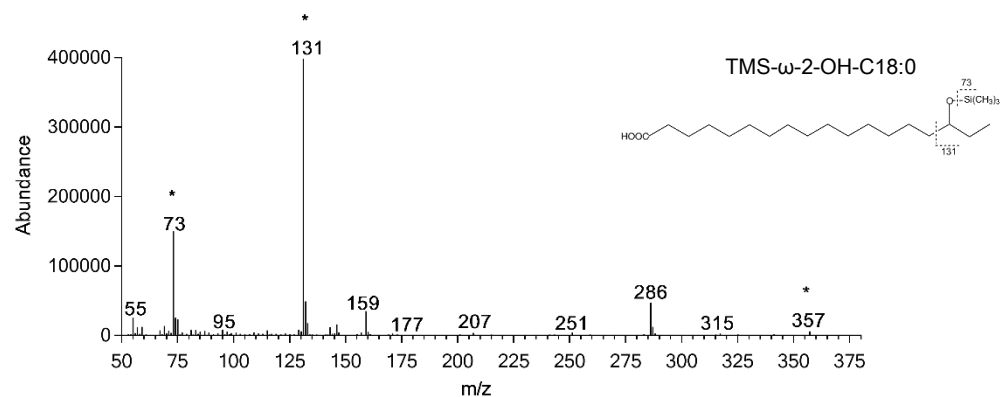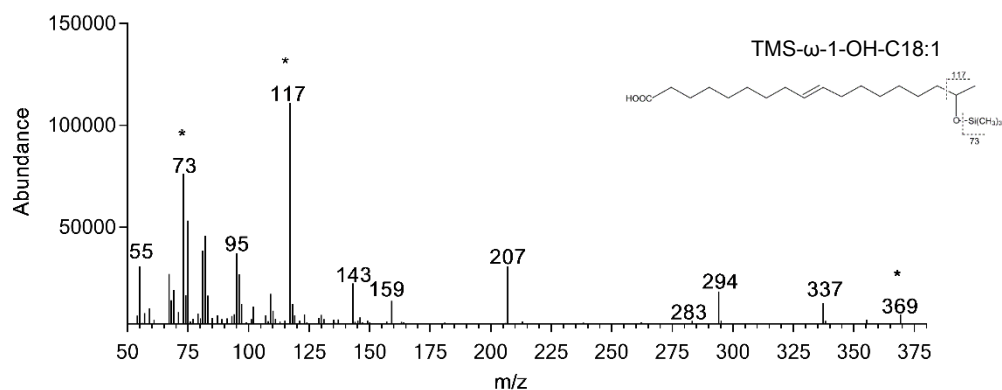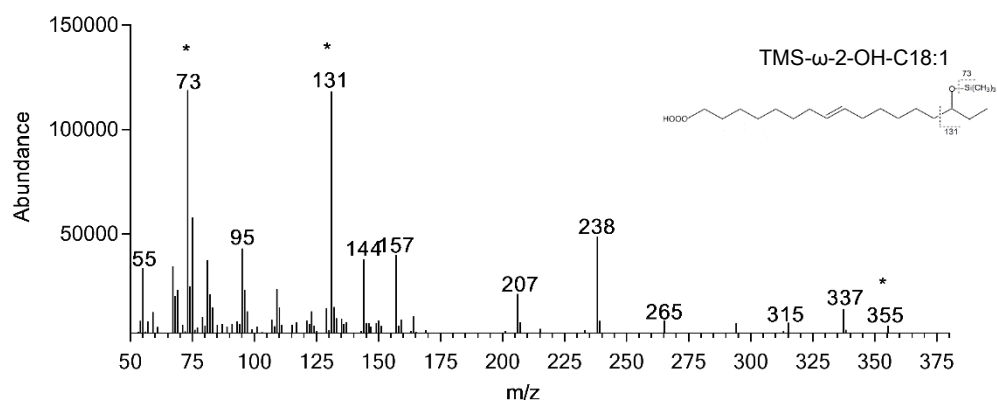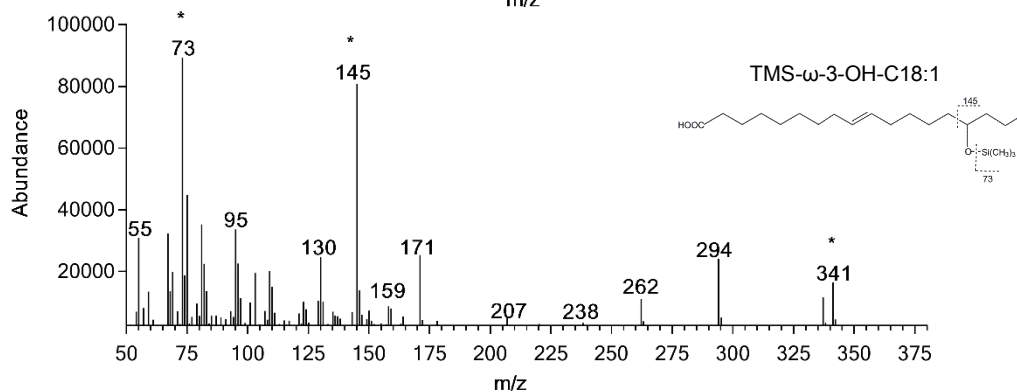

Supplement: Figure S4 — The chemical structure and mass spectra of HFA TMS derivatives from strain CPCc. Mass spectra of 3-OH-C14:0 and 3-OH-C14:1 TMS derivatives were same as Figure S1. *Indicates the characteristic mass ions. [file Image4.pdf]

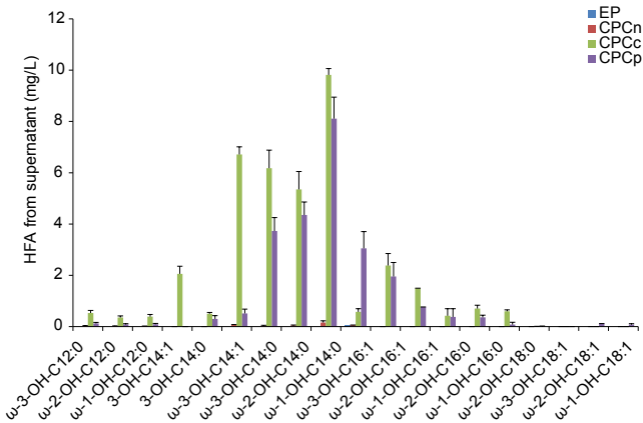

Supplement: Figure S5 — Hydroxyl fatty acid profile for recombinant strain EP, CPCn, CPCc, and CPCp, respectively. [file Image5.PDF]

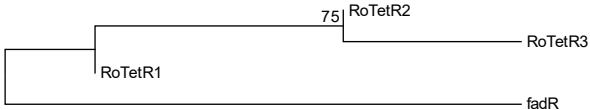

1

Supplement: Figure S6 — Phylogeny tree of three potential fatty acid metabolism regulators RoTetRs and FadR from E. coli. [file Image6.PDF]

**A**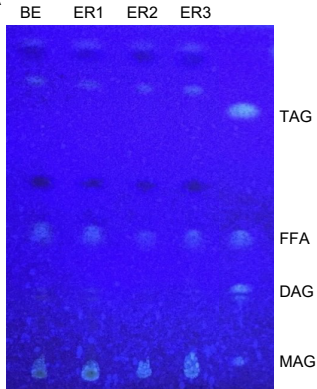**B**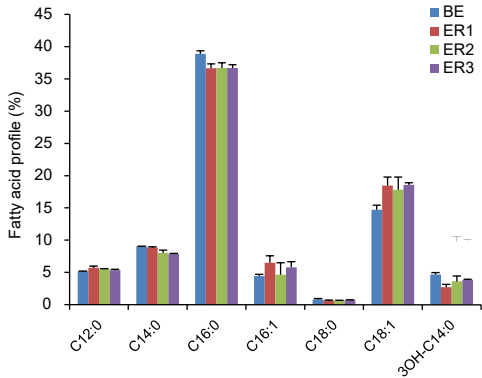

Supplement: Figure S7 — Lipid profile of recombinant E. coli hells expressing individual RoTetRs. (A) TLC showing the neutral lipids; (B) fatty acid profile. [file Image7.PDF]

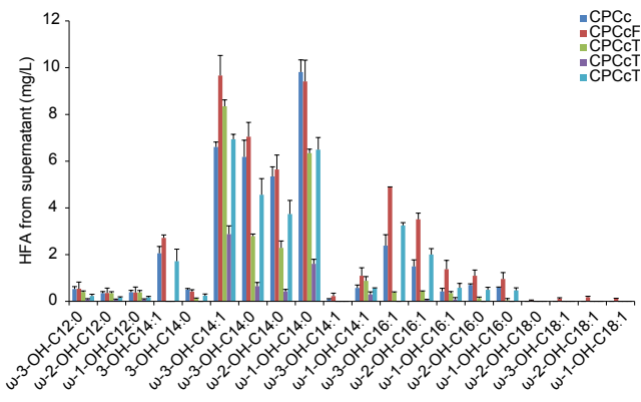

Supplement: Figure S8 — Hydroxyl fatty acid profile for recombinant strain CPCc, CPCcF, CPCcT1, CPCcT2, and CPCcT3, respectively. [file Image8.PDF]

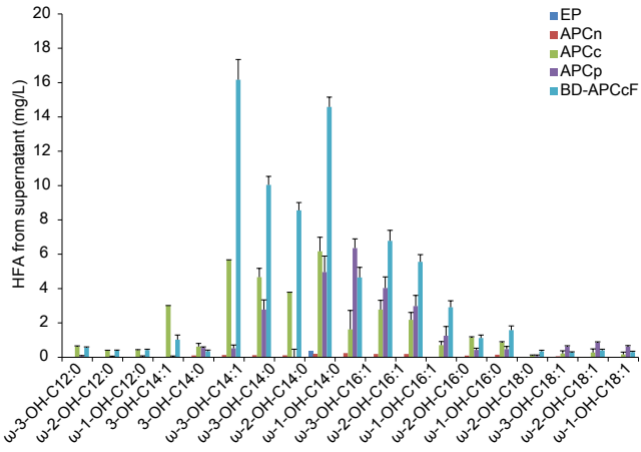

Supplement: Figure S9 — Hydroxyl fatty acid profile for recombinant strain EP, APCn, APCc, APCp, and BD-APCcF, respectively. [file Image9.PDF]
