# Supplementary material for: Metabolic Engineering for Enhanced Medium Chain Omega Hydroxy Fatty Acid Production in Escherichia coli
Source: Front Microbiol. 2018 Feb 7;9:139. doi: 10.3389/fmicb.2018.00139 (PMC5808347; doi:10.3389/fmicb.2018.00139)
Supplement: Table S1 — Strains and plasmids used in this study. [file Table1.DOCX]

**Table S1.** Strains and plasmids used in this study.

| Plasmid/strain | Description | Source |
| --- | --- | --- |
| **Plasmid** |  |  |
| pET28a(+) | Kan^r^ ori pBR322 lacl T7 | Novagen |
| pCDFDuet-1 | Str^r^ ori CDF lacl T7 | Novagen |
| pACYCDuet-1 | Cm^r^ ori P15A lacI T7 | Novagen |
| pEP | pET28a(+) carrying *P450_BM3_* from *B. megaterium* | This study |
| pECn | pET28a(+) carrying *CnFatB3* from *C. nucifera* | This study |
| pECc | pET28a(+) carrying *CcFatB1* from *C. camphora* | This study |
| pECp | pET28a(+) carrying *CpFatB2* from *C. lanceolata* | This study |
| pECt | pET28a(+) carrying *CtFatB* from *Cl. tetani E88* | This study |
| pELp | pET28a(+) carrying *LpFatB* from *L. phytofermentans ISDg* | This study |
| pEF | pET28a(+) carrying *fadR* from *E. coli MG1655* | This study |
| pER1 | pET28a(+) carrying *RoTetR1* from *R. opacus* PD630 | This study |
| pER2 | pET28a(+) carrying *RoTetR2* from *R. opacus* PD630 | This study |
| pER3 | pET28a(+) carrying *RoTetR3*from *R. opacus* PD630 | This study |
| pCPCn | pCDFDuet-1 carrying *P450_BM3_* and *CnFatB3* | This study |
| pCPCc | pCDFDuet-1 carrying *P450_BM3_* and *CcFatB1* | This study |
| pCPCp | pCDFDuet-1 carrying *P450_BM3_* and *CpFatB2* | This study |
| pAPCn | pACYCDuet-1 carrying *P450_BM3_* and *CnFatB3* | This study |
| pAPCc | pACYCDuet-1 carrying *P450_BM3_* and *CcFatB1* | This study |
| pAPCp | pACYCDuet-1 carrying *P450_BM3_* and *CpFatB2* | This study |
| pAPCc-1 | pACYCDuet-1 carrying *P450_BM3_* –RBS- *CcFatB1* in MCS1 | This study |
| pCPCc-1 | pCDFDuet-1 carrying *P450_BM3_* –RBS- *CcFatB1* in MCS1 | This study |
| **Strain** |  |  |
| DH5α | F^-^,φ80d*lacZ* ΔM15, Δ(*lac*ZYA -*arg*F )U169, *deo*R, *rec*A1, *end*A1, *hsd*R17 (rK^-^, mK^+^), *pho*A, *sup*E44, *λ^-^*, *thi*-1, *gyr*A96, *rel*A | Takara Bio |
| BL21 | *E. coli* BL21(DE3), *F- ompT gal dcm lon hsdSB(rB-mB-)λ(DE3)* | Takara Bio |
| BL21*ΔfadD* | Knockout of *fadD* encoding acyl-CoA synthetase in BL21 | This study |
| BE | *E. coli* BL21(DE3) carring pET28a(+) | This study |
| EP | *E. coli* BL21(DE3) carring pEP | This study |
| Cn | *E. coli* BL21(DE3) carring pECn | This study |
| Cc | *E. coli* BL21(DE3) carring pECc | This study |
| Cp | *E. coli* BL21(DE3) carring pECp | This study |
| Ct | *E. coli* BL21(DE3) carring pECt | This study |
| Lp | *E. coli* BL21(DE3) carring pELp | This study |
| CPCn | *E. coli* BL21(DE3) carring pCPCn | This study |
| CPCc | *E. coli* BL21(DE3) carring pCPCc | This study |
| CPCp | *E. coli* BL21(DE3) carring pCPCp | This study |
| CPCcF | *E. coli* BL21(DE3) carring pCPCc and pEF | This study |
| ER1 | *E. coli* BL21(DE3) carring pER1 | This study |
| ER2 | *E. coli* BL21(DE3) carring pER2 | This study |
| ER3 | *E. coli* BL21(DE3) carring pER3 | This study |
| CPCcR1 | *E. coli* BL21(DE3) carring pCPCc and pER1 | This study |
| CPCcR2 | *E. coli* BL21(DE3) carring pCPCc and pER2 | This study |
| CPCcR3 | *E. coli* BL21(DE3) carring pCPCc and pER3 | This study |
| APCn | *E. coli* BL21(DE3) carring pAPCn | This study |
| APCc | *E. coli* BL21(DE3) carring pAPCc | This study |
| APCp | *E. coli* BL21(DE3) carring pAPCp | This study |
| APCcF | *E. coli* BL21 (DE3) carring pAPCc and pEF | This study |
| *BD-*APCcF | *E. coli* BL21 (DE3)*ΔfadD* carring pAPCc and pEF | This study |
| *BD-*CPCcF | *E. coli* BL21 (DE3)*ΔfadD* carring pCPCc and pEF | This study |
| *BD-*APCc-1 | *E. coli* BL21 (DE3)*ΔfadD* carring pAPCc-1 | This study |
| *BD-*CPCc-1 | *E. coli* BL21 (DE3)*ΔfadD* carring pCPCc-1 | This study |
