# Supplementary material for: Metabolic Engineering for Enhanced Medium Chain Omega Hydroxy Fatty Acid Production in Escherichia coli
Source: Front Microbiol. 2018 Feb 7;9:139. doi: 10.3389/fmicb.2018.00139 (PMC5808347; doi:10.3389/fmicb.2018.00139)
Supplement: Table S2 — Details of primer sequences used in this test. [file Table2.docx]

**Supplementary** **Table 2.** List of primers used in this study.

| Primers | Nucleotide sequence |
| --- | --- |
| *P450*_BM3_-F | 5’-GGAATTCATGACAATTAAAGAAATGCCTCAG-3’ |
| *P450*_BM3_-R | 5’-CCGCTCGAGTCCCAGCCCACACGTCTTTTG-3’ |
| *CnFatB3*-F | 5’- GGAATTCATGGCTACCACCTCTCTGGC -3’ |
| *CnFatB3*-R | 5’- CCGCTCGAGAACAGAAGATTCAGCCGGGA -3’ |
| *CcFatB1*-F | 5’- GGAATTCATGGCTACCACCTCTCTGGC -3’ |
| *CcFatB1*-R | 5’- CCGCTCGAGAACAGAAGATTCAGCCGGGA -3’ |
| *CpFatB2*-F | 5’- GGAATTCATGGTTGCTGCTGCTGCCAGCGCTG-3’ |
| *CpFatB2*-R | 5’- CCGCTCGAGAGAGATAGAGTTACCGTTAGAGGT-3’ |
| *CtFat*-F | 5’- GGAATTCATGTCTAAAATGGAAAAAGACTACGA-3’ |
| *CtFat*-R | 5’- CCGCTCGAGGTTTTCAGCTTCTTTGTTTTTCTGCC-3’ |
| *LpFat*-F | 5’- GGAATTCATGTACTCTTTCGACTCTCGTGTTCG-3’ |
| *LpFat*-F | 5’- CCGCTCGAGAACAGTGGTACCGTAGAACGCAAC-3’ |
| *fadR-*F | 5’- CGGGATCCG ATGGTCATTAAGGCGCAAAGCCCG-3’ |
| *fadR-*R | 5’-CCGCTCGAGTCGCCCCTGAATGGCTAAATCACC-3’ |
| *RhTetR1-*F | 5’-GGATCCATGAACCCGCCCGTCACGAGTACC-3’ |
| *RhTetR1-*R | 5’-CAAGCTTGGGGGTGCTTGCGGCGACGGGAC-3’ |
| *RhTetR2-*F | 5’-GGATCCATGGACCGCAACCGGAAAATCCTCG-3’ |
| *RhTetR2-*R | 5’-CAAGCTTGGG CGACTTGCGGAAAGCGTCCAGGC-3’ |
| *RhTetR3-*F | 5’-GGATCCATGGCGAAGGACACGTCCGCCGTTC-3’ |
| *RhTetR3-*R | 5’-CAAGCTTGGG CTCCGTCAGGTGTCGGTACTTC-3’ |
