# Supplementary material for: Metabolic Engineering for Enhanced Medium Chain Omega Hydroxy Fatty Acid Production in Escherichia coli
Source: Front Microbiol. 2018 Feb 7;9:139. doi: 10.3389/fmicb.2018.00139 (PMC5808347; doi:10.3389/fmicb.2018.00139)
Supplement: Table S3 — Fatty acid profile of cellular total fatty acid (TFA) produced by BL21(DE3) harboring individual plant acyl-ACP thioesterase gene. [file Table3.DOCX]

Table S3 Fatty acid profile of total fatty acid (TFA) produced by BL21(DE3) harboring individual plant acyl-ACP thioesterase gene.

| Strain/medium | Fatty acid profile (%) | | | | | | | | | | |
| --- | --- | --- | --- | --- | --- | --- | --- | --- | --- | --- | --- |
|  | 12:0 | 14:0 | | | 14:1 | 16:0 | 16:1 | 18:0 | 18:1 | 3-OH C14:0 | others |
| **LB medium** | | | | | | | | | | |  |
| BE^a^ | 4.9±0.2 | 7.5±0.1 | | | 0.0±0.1 | 37.8±0.5 | 7.3±0.1 | 0.9±0.1 | 16.1±0.3 | 10.5±0.1 | 15.0±0.2 |
| Cn^a^ | 5.2±0.1 | 7.8±0.2 | | | 0.1±0.0 | 32.1±0.5 | 13.2±0.1 | 0.8±0.1 | 22.5±1.0 | 9.9±0.1 | 8.3±0.8 |
| Cc^a^ | 4.8±0.1 | 8.4±0.6 | | | 0.1±0.0 | 29.8±0.5 | 16.3±0.7 | 0.7±0.0 | 25.8±1.7 | 9.0±0.4 | 5.2±0.6 |
| Cp^a^ | 6.0±0.4 | 7.8±0.3 | | | 0.0±0.0 | 32.7±1.3 | 6.8±0.9 | 0.8±0.1 | 20.3±1.3 | 13.1±0.3 | 12.6±1.1 |
| **LB medium + 0.5% glucose** | | | | | | | | | | |  |
| BE | 4.2±0.1 | 7.5±0.1 | | | 0.2±0.0 | 50.3±0.1 | 6.7±0.1 | 1.1±0.1 | 10.0±0.3 | 7.3±0.1 | 12.7±0.2 |
| Cn | 8.5±0.2 | 22.2±0.1 | | | 2.9±0.3 | 26.9±0.6 | 11.8±0.6 | 0.5±0.0 | 16.6±0.3 | 6.9±0.2 | 3.7±0.3 |
| Cc | 3.6±0.1 | 13.4±0.5 | | | 5.7±0.2 | 32.6±0.2 | 13.7±0.5 | 0.8±0.0 | 16.8±0.1 | 7.2±0.3 | 6.3±0.3 |
| Cp | 5.0±0.1 | 30.2±0.6 | | | 3.5±0.3 | 5.0±1.3 | 24.1±0.1 | 0.1±0.0 | 10.1±0.2 | 19.9±1.3 | 2.0±0.1 |
| **M9 medium + 0.5% glucose** | | | | | | | | | | |  |
| BE | 4.8±0.0 | | 8.5±0.4 | | 0.1±0.1 | 50.5±0.4 | 2.3±0.0 | 0.7±0.0 | 2.3±0.1 | 6.2±0.1 | 24.5±0.4 |
| Cn | 8.9±0.6 | | 17.9±0.3 | | 9.4±0.5 | 19.8±1.1 | 20.2±0.3 | 0.2±0.0 | 13.2±0.2 | 7.5±1.1 | 2.9±0.2 |
| Cc | 3.6±0.0 | | | 22.4±0.0 | 11.0±0.3 | 23.9±0.3 | 15.7±0.2 | 0.4±0.0 | 14.0±0.2 | 3.4±0.1 | 5.6±0.1 |
| Cp | 2.8±0.3 | | 18.7±0.7 | | 2.5±0.5 | 14.7±1.4 | 27.5±2.1 | 0.2±0.0 | 6.4±1.2 | 10.9±1.7 | 16.4±1.8 |

^a^BE: BL21(DE3); Cn: BL21(DE3) harboring *CnFatB3;* Cc: BL21(DE3) haroboring *CcFatB1*; Cp: BL21(DE3) harboring *CpFatB2*;
