# Supplementary material for: Metabolic Engineering for Enhanced Medium Chain Omega Hydroxy Fatty Acid Production in Escherichia coli
Source: Front Microbiol. 2018 Feb 7;9:139. doi: 10.3389/fmicb.2018.00139 (PMC5808347; doi:10.3389/fmicb.2018.00139)
Supplement: Table S4 — Fatty acid profile of cellular cell free fatty acid (FFA) a produced by BL21(DE3) harboring individual plant acyl-ACP thioesterase gene. [file Table4.DOCX]

Table S4 Fatty acid profile of cell free fatty acid (FFA) ^a^ produced by BL21(DE3) harboring individual plant acyl-ACP thioesterase gene.

| Strain/medium | Fatty acid profile (%) | | | | | | | |
| --- | --- | --- | --- | --- | --- | --- | --- | --- |
|  | 12:0 | 14:0 | 14:1 | 16:0 | 16:1 | 18:0 | 18:1 | others |
| **LB medium** | | | | | | | | |
| BE^b^ | 6.0±0.6 | 8.9±1.3 | 0.0±0.0 | 57.0±2.7 | 0.5±0.1 | 23.9±1.9 | 0.8±0.3 | 3.0±0.3 |
| Cn^b^ | 4.3±1.0 | 7.6±0.6 | 2.2±0.7 | 45.3±1.8 | 6.2±0.5 | 11.5±1.1 | 13.6±0.2 | 9.2±0.8 |
| Cc^b^ | 2.8±0.5 | 8.5±1.1 | 1.8±0.0 | 41.8±4.8 | 10.7±1.0 | 7.4±0.7 | 20.7±0.6 | 6.3±0.6 |
| Cp^b^ | 2.8±0.2 | 21.6±0.2 | 4.0±0.0 | 19.4±4.8 | 31.7±0.5 | 2.2±0.4 | 15.0±0.3 | 3.5±0.1 |
| **LB medium + 0.5% glucose** | | | | | | | | |
| BE | 0.9±0.1 | 5.8±0.1 | 0.3±0.0 | 68.2±1.2 | 6.0±0.7 | 2.0±0.1 | 9.5±0.9 | 7.3±0.1 |
| Cn | 11.1±0.2 | 34.3±1.3 | 4.5±0.3 | 24.4±0.4 | 12.9±0.9 | 0.4±0.0 | 11.8±0.6 | 0.7±0.1 |
| Cc | 1.8±0.1 | 19.1±0.5 | 9.5±0.6 | 30.3±0.5 | 19.9±0.4 | 1.0±0.1 | 15.8±0.4 | 2.7±0.1 |
| Cp | 6.3±0.3 | 46.3±0.9 | 5.8±0.2 | 1.8±0.2 | 35.3±0.8 | 0.0±0.0 | 4.5±0.1 | 0.1±0.0 |
| **M9 medium + 0.5% glucose** | | | | | | | | |
| BE | 5.3±0.1 | 7.7±0.8 | 1.8±0.4 | 47.1±1.9 | 0.3±0.1 | 36.2±1.5 | 1.2±0.2 | 0.3±0.0 |
| Cn | 11.8±0.9 | 25.4±0.1 | 13.2±0.1 | 14.0±0.1 | 23.8±1.1 | 0.2±0.0 | 11.3±0.2 | 0.1±0.0 |
| Cc | 4.2±0.1 | 37.5±0.5 | 19.1±1.1 | 13.1±0.8 | 18.8±0.3 | 0.3±0.0 | 6.5±0.2 | 0.5±0.0 |
| Cp | 3.3±0.2 | 30.8±0.3 | 7.3±0.5 | 3.0±0.6 | 44.0±0.7 | 0.0±0.0 | 10.7±0.4 | 0.9±0.1 |

^a^FFA: free fatty acids from cell lysate;

^b^BE: BL21(DE3); Cn: BL21(DE3) harboring *CnFatB3;* Cc: BL21(DE3) haroboring *CcFatB1*; Cp: BL21(DE3) harboring *CpFatB2*.
